# Supplementary material for: LncRNA UCA1, Upregulated in CRC Biopsies and Downregulated in Serum Exosomes, Controls mRNA Expression by RNA-RNA Interactions
Source: Mol Ther Nucleic Acids. 2018 Jun 2;12:229–41. doi: 10.1016/j.omtn.2018.05.009 (PMC6023947; doi:10.1016/j.omtn.2018.05.009)
Supplement: Document S1. Figures S1–S3 and Tables S1–S6, S9, and S10 [file mmc1.pdf]

OMTN, Volume *12*

Supplemental Information

LncRNA UCA1, Upregulated in CRC Biopsies and Downregulated in Serum Exosomes, Controls mRNA Expression by RNA-RNA Interactions

Cristina Barbagallo, Duilia Brex, Angela Caponnetto, Matilde Cirnigliaro, Marina Scalia, Antonio Magnano, Rosario Caltabiano, Davide Barbagallo, Antonio Biondi, Alessandro Cappellani, Francesco Basile, Cinzia Di Pietro, Michele Purrello, and Marco Ragusa

Supplemental Figure 1: Expression of lncRNAs and circRNAs in CRC cell lines HCT-116 and Caco-2.

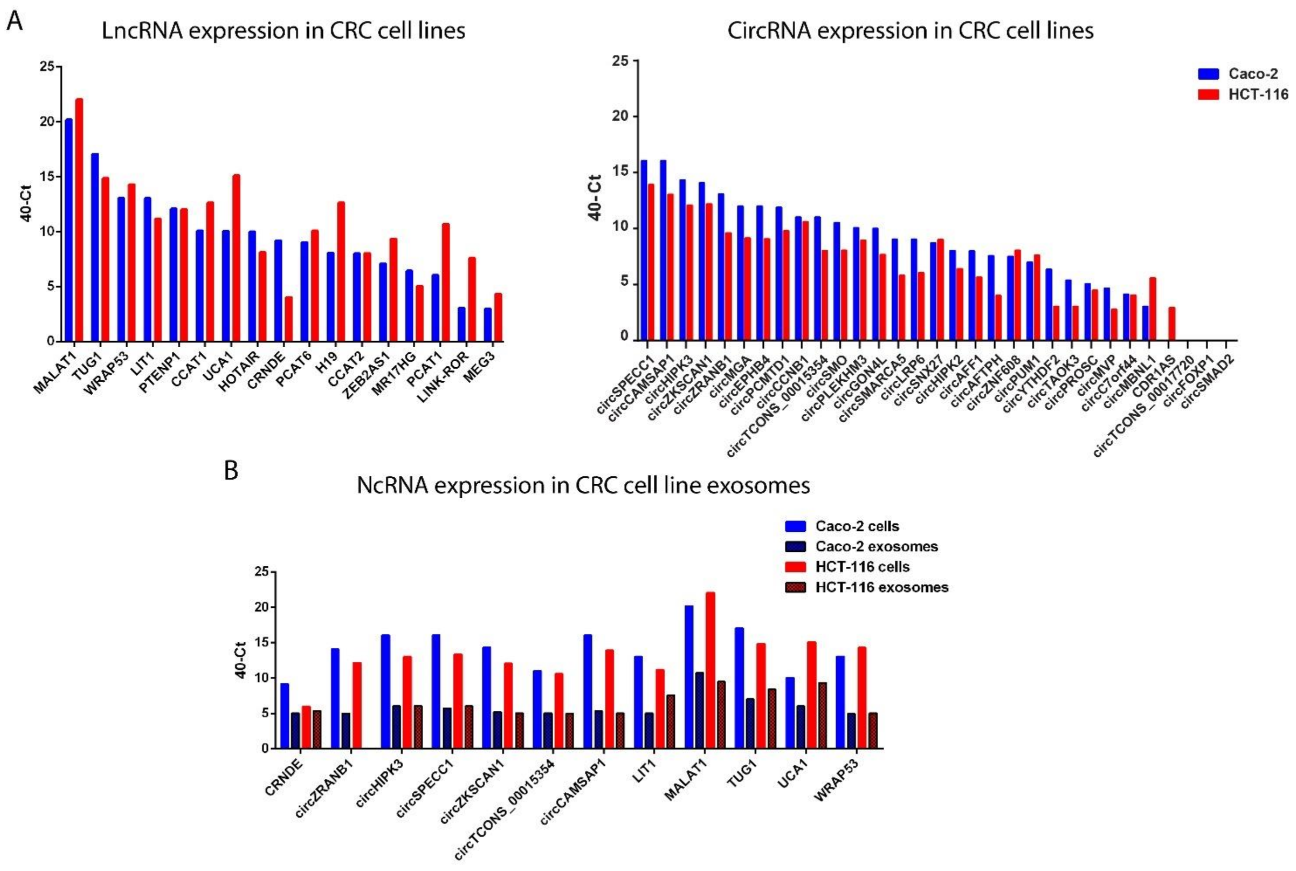

The most abundant ncRNAs in CRC cells were investigated in their exosomes. Expression is shown as 40-Ct.

Supplemental Figure 2: Effects of MAPK inhibition.

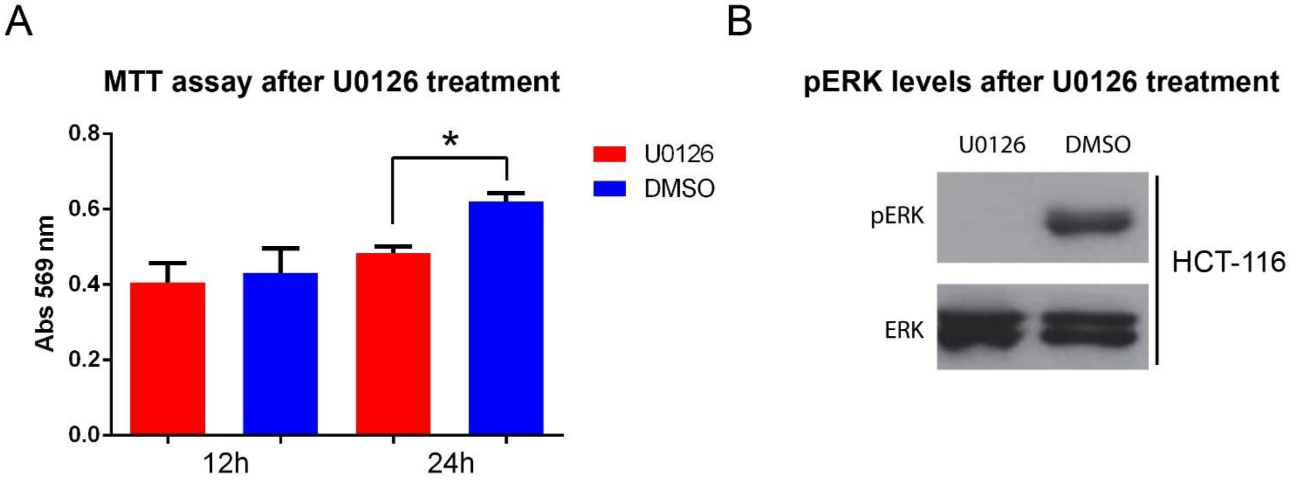

A) treatment with U0126 significantly reduced cell viability, measured by MTT assay 24 hours after treatment; cells treated with DMSO were used as controls; error bars represent standard deviation. *: p-value = 4.96E-7. B) Western Blot showing Erk and p-Erk levels in HCT-116 cells treated with MAPK inhibitor U0126 compared to control cells treated with DMSO at 12 hours post treatment: MAPK inhibition is showed by reduced levels of p-Erk compared to Erk.

Supplemental Figure 3: Biological processes controlled by the ceRNA network regulated by UCA1.

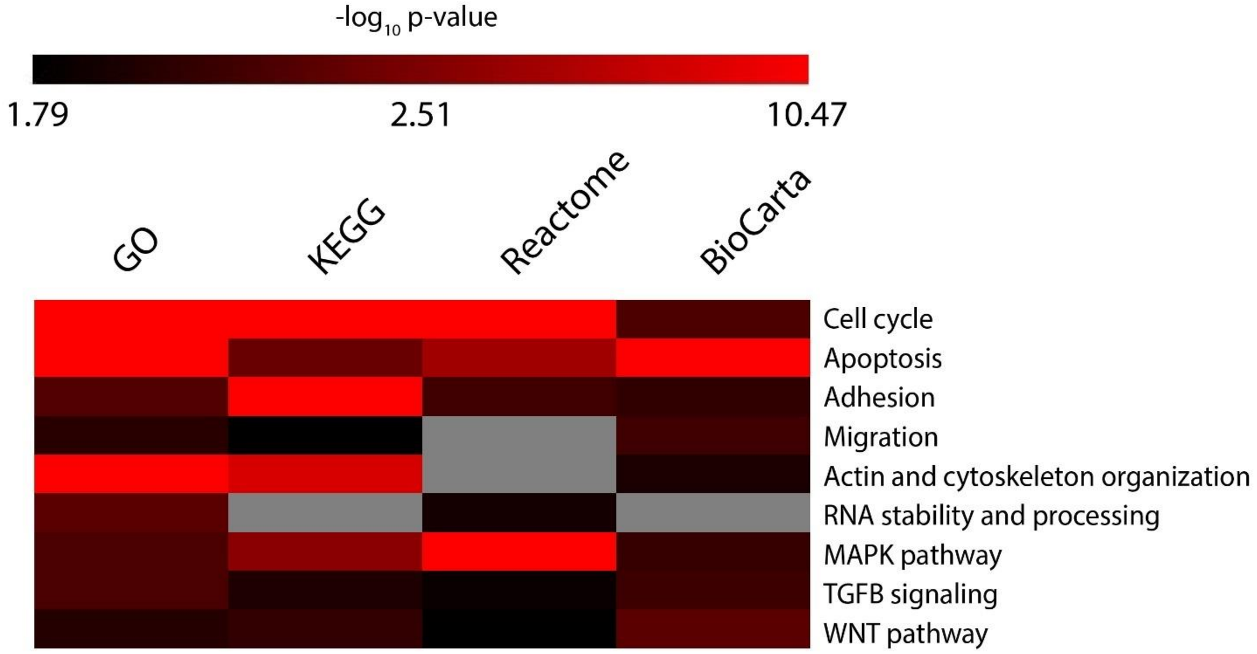

Over-represented biological functions of the molecular network arose from interactions among ANLN, BIRC5, IPO7, KIF23, and KIF2A, retrieved from different annotation databases (GO, KEGG, Reactome, BioCarta). Data are shown as -log10 of p-values for each biological process, as shown in the colour coded bar.

Supplemental Table 1: Analysis of CRC GEO datasets.

	**GEO dataset ID**	**Pearson (p-value)**
MALAT1	GSE37892	-0.475 (1.10E-08)
	GSE35896	-0.4 (1.30E-03)
	GSE8671	-0.33 (7.70E-03)
	GSE13294	-0.24 (2.70E-03)
	GSE4554	-0.23 (3.00E-02)
	GSE33114	-0.217 (2.00E-02)
	GSE14333	0.477 (6.80E-18)
	GSE23878	0.523 (2.20E-05)
	GSE39395	0.616 (1.30E-03)
	GSE3629	0.95 (2.50E-62)
UCA1	GSE33114	-0.272 (4.40E-03)
	GSE14333	-0.18 (2.10E-03)
	GSE2109	0.375 (2.00E-02)
	GSE8671	0.578 (5.80E-07)
	GSE3629	0.69 (9.90E-19)

Expression correlation between ncRNAs deregulated at 6 hours after U0126 treatment and HSPA5, a transcriptional target of p-Erk. The Pearson coefficient and its p-value (between brackets) are shown.

Supplemental Table 2: Hypothetical transcription factors regulating UCA1 expression.

	**CEBPB**	**TEAD4**	**TFAP2A**	**TFAP2C**
**TFBS Position**	chr19:15938912-15939187	chr19:15939692-15939850	chr19:15939115-15939748	chr19:15939109-15939583
**Size (bp)**	276	159	634	475
**Position from TSS (bp)**	-845	-65	-642	-648
**GSE8671**	FC **1.74 (<0.00001)**	**3.5 (<0.00001)**	**1.5 (0.01)**	**2.24 (0.0002)**
	**0.65 (<0.00001)**	**0.7 (<0.00001)**	**0.5 (0.00002)**	0.15 (0.22)
**GSE9348**	**1.19 (<0.00001)**	**1.53 (<0.00001)**	**1.31 (0.00009)**	1.08 (0.23)
	**0.26 (0.02)**	**0.25 (0.01)**	0.18 (0.1)	0.09 (0.4)
**GSE20842**	**1.15 (<0.00001)**	**1.2 (<0.00001)**	**1.07 (<0.00001)**	**1.01 (<0.00001)**
	**0.4 (<0.00001)**	**0.61 (<0.00001)**	**0.59 (<0.00001)**	**0.37 (<0.00001)**
**GSE20916**	**1.1 (<0.00001)**	**1.71 (<0.00001)**	**1.01 (0.01)**	**1.02 (0.002)**
	**0.32 (0.0006)**	**0.35 (0.0001)**	**0.25 (0.007)**	**0.22 (0.02)**
**TCGA**	**3.08 (<0.00001)**	**3.62 (<0.00001)**	**2.24 (<0.00001)**	**2.33 (<0.00001)**
	**0.23 (0.001)**	**0.24 (0.001)**	**0.26 (0.0004)**	**0.20 (0.005)**

For each TFB, the genomic position, the size of the bound region and its distance from the transcription start site (TSS) are shown. The expression of TFs was investigated in CRC datasets showing increased expression of UCA1 in tumor tissues compared with normal mucosa. For each gene, fold change (FC), Pearson coefficient (Pc), and their p-value (between brackets) are shown. Statistically significant results are highlighted in bold.

Supplemental Table 3: Expression of miRNAs hypothetically sponged by UCA1 in CRC tissues compared to normal mucosa from GEO datasets.

	**GSE108153**	**GSE38389**	**GSE41655**	**GSE73487**	**GSE30454**	**GSE18392**	**GSE10259**	**GSE53339**
**miR-135a-5p**	1 (0.48)	**-1.01 (0.01)**	-1.17 (0.16)	1.06 (0.17)	**-3.28 (4.15E-10)**	**-1.4 (0.04)**		-1.22 (0.19)
**miR-143-3p**	**-4.77 (0.006)**	**-1.78 (5.77E-06)**	**-2.6 (1-79E-11)**	**-1.61 (0.002)**	**-1.21 (2.11E-05)**	1 (0.21)	**-2.45 (0.0009)**	**-13.61 (0.007)**
**miR-214-3p**	**1.28 (0.05)**	1 (0.4)	**-1.81 (0.003)**	**-1.33 (0.008)**	**-1.19 (0.01)**	1 (0.46)	1 (0.43)	**-1.17 (0.05)**
**miR-1271-5p**	**-1.54 (0.006)**	1 (0.36)		-1.09 (0.1)	**-1.81 (0.001)**			

Fold change and p-value (between brackets) are shown. Statistically significant results are highlighted in bold.

Supplemental Table 4: miRNAs and mRNAs selected as hypothetical targets of UCA1.

miRNA targets	miR-135a (2)	miR-143 (4)	miR-214 (2)	miR-1271 (1)
**ANLN**	x		x	x
**BIRC5**	x	x		
**BZW2**	x			
**CD46**	x	x	x	
**DEK**	x		x	
**DNMT3A**		x		
**HMMR**		x		x
**IPO7**			x	
**KIF2A**				x
**KIF23**	x	x		x
**MACC1**		x		
**MMP7**		x		
**MYC**	x			
**TRIP13**	x			

The number of miRNA binding sites lying within UCA1 sequence is shown for each miRNA between brackets; mRNA targets of each miRNA are indicated with "x".

Supplemental Table 5: **Expression of TFs and miRNA targets in CRC datasets.**

	**GSE8671**		**GSE9348**		**GSE20842**		**GSE20916**		**TCGA**	
	**FC (p-val.)**	**Pc (p-val.)**	**FC (p-val.)**	**Pc (p-val.)**	**FC (p-val.)**	**Pc (p-val.)**	**FC (p-val.)**	**Pc (p-val.)**	**FC (p-val.)**	**Pc (p-val.)**
ANLN	**4.3 (<0.00001)**	**0.51 (0.00001)**	**1.3 (<0.00001)**	0.18 (0.1)	**1.23 (<0.00001)**	**0.63 (0.00001)**	**1.55 (<0.00001)**	**0.29 (0.002)**	**2.24 (<0.00001)**	**0.23 (0.002)**
BIRC5	**3.12 (<0.00001)**	**0.51 (0.00001)**	**1.24 (<0.00001)**	**0.23 (0.03)**	\	\	**1.41 (<0.00001)**	**0.28 (0.003)**	**1.85 (<0.00001)**	**0.19 (0.008)**
BZW2	**1.57 (<0.00001)**	**0.46 (0.0001)**	**1.07 (<0.00001)**	0.1 (0.33)	**1.05 (<0.00001)**	**0.56 (< 0.00001)**	**1.07 (<0.00001)**	**0.32 (0.0006)**	**2.2 (<0.00001)**	**0.37 (<0.00001)**
CD46	**1.38 (<0.00001)**	**0.39 (0.001)**	**1.03 (0.0001)**	0.15 (0.17)	1 (0.12)	0.10 (0.22)	**1.07 (<0.00001)**	**0.4 (0.00002)**	**3.2 (<0.00001)**	**0.19 (0.01)**
CEBPB	**1.74 (<0.00001)**	**0.65 (<0.00001)**	**1.19 (<0.00001)**	**0.26 (0.02)**	**1.15 (<0.00001)**	**0.4 (<0.00001)**	**1.1 (<0.00001)**	**0.32 (0.0006)**	**3.08 (<0.00001)**	**0.23 (0.001)**
DEK	**1.39 (<0.00001)**	**0.58 (<0.00001)**	**1.04 (0.0007)**	0.12 (0.2)	1.05 (<0.00001)	0.511 (<0.00001)	1.06 (<0.00001)	0.26 (0.007)	2.64 (<0.00001)	0.11 (0.14)
DNMT3A	**1.68 (<0.00001)**	**0.37 (0.002)**	**1.2 (<0.00001)**	0.13 (0.22)	**1.05 (<0.00001)**	**0.37 (<0.00001)**	**1.13 (<0.00001)**	**0.38 (0.008)**	**1.15 (0.05)**	**0.15 (0.04)**
HMMR	**2.26 (<0.00001)**	**0.41 (0.0006)**	**1.15 (<0.00001)**	0.08 (0.44)	**1.07 (<0.00001)**	**0.45 (<0.00001)**	**1.18 (<0.00001)**	**0.22 (0.02)**	**1.97 (<0.00001)**	**0.27 (0.0002)**
IPO7	**2.02 (<0.00001)**	**0.57 (<0.00001)**	**1.06 (<0.00001)**	0.34 (0.25)	**1.03 (<0.00001)**	**0.23 (0.006)**	**1.1 (<0.00001)**	**0.34 (0.0003)**	**1.31 (0.0005)**	0.08 (0.25)
KIF23	**3.49 (<0.00001)**	**0.51 (0.00001)**	**1.15 (<0.00001)**	0.04 (0.69)	**1.11 (<0.00001)**	**0.47 (<0.00001)**	**1.45 (<0.00001)**	0.17 (0.07)	**1.74 (<0.00001)**	0.12 (0.11)
KIF2A	**1.26 (<0.00001)**	**0.3 (0.01)**	1.01 (0.38)	0.1 (0.37)	**1.02 (0.004)**	**0.34 (0.00007)**	**1.05 (0.001)**	0.12 (0.19)	**1.38 (0.001)**	0.09 (0.19)
MACC1	**1.9 (<0.00001)**	**0.35 (0.004)**	**1.34 (<0.00001)**	**0.27 (0.001)**	**1.17 (<0.00001)**	**0.58 (<0.00001)**	**1.34 (<0.00001)**	**0.35 (0.0002)**	**3.4 (<0.00001)**	**0.32 (0.00001)**

MMP7	**40 (0.00001)**	**0.27 (0.02)**	**1.64 (<0.00001)**	**0.37 (0.0006)**	**1.74 (<0.00001)**	**0.69 (<0.00001)**	**3.39 (<0.00001)**	0.47 (0.47)	**6.47 (<0.00001)**	**0.43 (<0.00001)**
MYC	**3.13 (<0.00001)**	**0.6 (< 0.00001)**	**1.22 (<0.00001)**	**0.35 (0.001)**	**1.06 (<0.00001)**	**0.54 (<0.00001)**	**1.21 (<0.00001)**	**0.36 (0.0001)**	**2.27 (<0.00001)**	**0.4 (<0.00001)**
TEAD4	**3.5 (<0.00001)**	**0.7 (<0.00001)**	**1.53 (<0.00001)**	**0.25 (0.01)**	**1.2 (<0.00001)**	**0.61 (<0.00001)**	**1.71 (<0.00001)**	**0.35 (0.0001)**	**3.62 (<0.00001)**	**0.24 (0.001)**
TFAP2A	**1.5 (0.01)**	**0.5 (0.00002)**	**1.31 (0.00009)**	0.18 (0.1)	**1.07 (<0.00001)**	**0.59 (<0.00001)**	**1.01 (0.01)**	**0.25 (0.007)**	**2.24 (<0.00001)**	**0.26 (0.0004)**
TFAP2C	**2.24 (0.0002)**	0.15 (0.22)	1.08 (0.23)	0.09 (0.4)	**1.01 (<0.00001)**	**0.37 (<0.00001)**	**1.02 (0.002)**	**0.22 (0.02)**	**2.33 (<0.00001)**	**0.20 (0.005)**
TRIP13	**3.59 (<0.00001)**	**0.52 (< 0.00001)**	**1.25 (<0.00001)**	**0.21 (0.05)**	**1.17 (<0.00001)**	**0.6 (<0.00001)**	**1.47 (<0.00001)**	**0.35 (0.0002)**	**3.56 (<0.00001)**	**0.33 (<0.00001)**
UCA1	**4.3 (<0.00001)**	\	**1.13 (0.002)**	\	**1.27 (<0.00001)**	\	**1.74 (<0.00001)**	\	**2.09 (<0.00001)**	\

TFs and miRNA targets of UCA1 showed increased expression in tumor tissues compared to normal mucosa. For each gene, fold change (FC), Pearson coefficient (Pc), and their p-value (between brackets) are shown. Statistically significant results are highlighted in bold.

Supplemental Table 6: **MiRNAs binding the 3'-UTRs of selected miRNA targets were predicted by microrna.org.**

	ANLN	BIRC5	IPO7	KIF2A	KIF23	Expression in CRC
**hsa-let-7a**				388		down
**hsa-let-7d**				388		down
**hsa-let-7g**				388		down
**hsa-miR-1**				656		down
**miR-1271**					325	down
**hsa-miR-128**			267			down
**hsa-miR-135a**	569	648			40	down
**hsa-miR-135b**		647			40	up
**hsa-miR-15a**	794		803			down
**hsa-miR-15b**	794		805	646		down
**hsa-miR-16**	794		804			down
**hsa-miR-182**	820					up
**hsa-miR-183**				601	197	up
**hsa-miR-186**			450			down
**hsa-miR-190**				607		down
**hsa-miR-192**			473			down
**hsa-miR-195**	796		805			down
**hsa-miR-206**				658		down
**hsa-miR-214**			3			down
**hsa-miR-215**			473			down
**hsa-miR-217**	642					up
**hsa-miR-218**	615	620, 642				down
**hsa-miR-22**			303			down
**hsa-miR-224**			732, 761			up
**hsa-miR-300**					180	up
**hsa-miR-31**	821					up
**hsa-miR-326**					130	down
**hsa-miR-330-5p**					128	down

**hsa-miR-340**			455			down
**hsa-miR-342-3p**	568					down
**hsa-miR-361-5p**			479			down
**hsa-miR-376c**	800					down
**hsa-miR-377**	568	748				down
**hsa-miR-381**					180	down
**hsa-miR-410**				667		down
**hsa-miR-424**	795		804			up
**hsa-miR-448**	644					down
**hsa-miR-495**	853					down
**hsa-miR-497**	795		807			down
**hsa-miR-503**	793		804			up
**hsa-miR-539**					32	down
**hsa-miR-613**				660		down
**hsa-miR-653**					168	down
**hsa-miR-873**		1100				down

For each miRNA, the position of the binding site(s) on 3’-UTR and its expression in CRC datasets are shown.

Supplemental Table 7: expression of miRNAs binding the 3'-UTR in CRC expression datasets.

Supplemental Table 8: Functional enrichment analysis of the ceRNA network regulated by UCA1.

Supplemental Table 9: PCR primers.

Gene	Forward primer	Reverse primer
**ANLN**	TGTCTTCGTGGCCGATTTGA	TTTGAGGGGAGTGCTCTGAC
**BIRC5**	GAGGCTGGCTTCATCCACTG	CCTTTGCATGGGGTCGTCAT
**BZW2**	TTGGAGAAGGCATTTGAAGATGAAA	TCGCCAACTTTGTCTGCTCT
**CCAT1**	GGAAAGGTGCCGAGACATGA	GCCATACAGAGCCAACCTGG
**CCAT2**	CCTGCAGAGGGCACTAGACT	CCAGGGTCAGGCAATTGGTC
**CD46**	GGTGTTGCTGCTGTACTCCT	ACCAATGAGCTCCATAGCTTCAA
**CRNDE**	TCGATCGCGCTATTGTCATGG	CGCCTCGCTTAGACATTGGC
**DEK**	CAGCACCACCAAGAAGAATCAA	ACTCTTCATCTGTAGGGGGTTTC
**DNMT3A**	CGACCCCACCGGCCATA	CTCTGGTGAACGGTGCCTCT
**GAPDH**	GGCTAGCTGGCCCGATTT	AGGCGCCCAATACGACCAAA
**H19**	GAGTCGGCACACTATGGCTG	GTCCGGATTCAAAGGCCCAG
**HMMR**	ATGGTGCAAAGCCTTGAAGATG	GGCCGCTTTTTCCTGTAATGA
**HOTAIR**	CCCGGAGGTGCTCTCAATCA	ACTCCCCTACTGCAGGCTTC
**IPO7**	CAGGAGAAGATGCAGAGTGTCA	TCTAAGGCTGCTTCCACGAAT
**KIF2A**	GGGAGTTCCCCTCAGAGAGA	TGTGAAACAGCTTCGTGGAAAG
**KIF23**	TGGATATCAGCATCACGCACA	CTTCTGGCATGGCTTTCGAG
**LINC01764**	CTCCGGACTGCTTCAAGTGT	GTTATTCACCGTCACAAGTTCTCA
**LINK-ROR**	CTTCCAGGTCTCAGGAATGGG	AGGTGGCTGGTGAGAGATCC
**LIT1**	CAGGACATGCCCACACTAGC	CGTTTCCTCTTACAGTGGCGG
**MACC1**	GCCACACAGAGAGAAATACAAGG	CGTGCGACTAACTCTTGGCA
**MALAT1**	TAGCTTGGATCCTTGTGGGC	AACCCACCAAAGACCTCGAC
**MEG3**	GCCAGCTGTCCCTCTTACCT	GCATAGCAAAGGTCAGGGCT
**MIR17HG**	TGCCACGTGGATGTGAAGATT	GTTCTCCAGGAAGTTGCAGGC
**MMP7**	AAGTGGTCACCTACAGGATCG	GGGATCTCTTTGCCCCACAT
**MYC**	CAGGACCCGCTTCTCTGAAA	CTAACGTTGAGGGGCATCGT
**PCAT1**	AGGCCAAGCTACAGGGAGAAA	TGGCATTATCTTGGGAGGTTCC

**PCAT6**	CTCTTGGACAACACTCCGCC	ATCTGGCGCTGCACAAATGA
**PPIA**	ACTGAGTGGTTGGATGGCAAGC	AGCGCTCCATGGCCTCCACA
**PTENP1**	CATTCTTGCATGTATTTGGGTTAGG	GGTATATGGTCCAGAGTCCAGC
**TRIP13**	AGCTGAATGAAGATGGCCCC	TACCAAGCTGTCCCAAAGCC
**TUG1**	CTTTCACCATGGGGTGCCTG	AGCTGCTTTACACTGGGTGC
**UCA1**	TGCACCCTAGACCCGAAACT	CAAGTGTGACCAGGGACTGC
**WRAP53**	ACCGGACTGTGCGTGTTTTT	CTGGGCTGAAGGCTATGCAG
**ZEB2AS1**	ACGCGCCACCTATCTTTGTG	TGCCCTAAGATGCAGCTCCC

PCR primers designed for selected lncRNAs, mRNAs and housekeeping genes.

Supplemental Table 10: GEO datasets.

GEO datasets	Samples
GSE10715	peripheral blood (19 tumors vs 11 controls), blood smears (10 tumors vs 10 controls)
GSE13294	fresh-frozen primary tumor tissues (74)
GSE14333	fresh-frozen primary tumor tissues (290)
GSE17538	fresh-frozen primary tumor tissues (232)
GSE18088	frozen or formalin-fixed and paraffin-embedded tissues (53)
GSE18105	cancer tissues and normal adjacent tissues (17), laser capture microdissected primary tumors (77)
GSE20916	distant full-thickness normal colon, normal colon mucosa dissected from tumor, adenoma, and carcinoma (40)
GSE2109	tumor tissues (315)
GSE21510	laser capture microdissected and homogenized tumors (148)
GSE23878	tumor tissues (35 tumors vs 24 matched controls)
GSE33114	fresh frozen or formalin-fixed and paraffin-embedded tissues (90 tumors vs 6 controls)
GSE35896	primary tumor tissues (62)
GSE3629	53 ulcerative colitis tissues (10 UC-associated cancer, 43 non-cancer), 60 sporadic cancer tissues
GSE39582	fresh-frozen primary tumor tissues (566)
GSE41258	primary colon adenocarcinomas, adenomas, metastasis and corresponding normal mucosae (390)

GEO datasets on CRC samples analysed to confirm DE lncRNA expression. For each dataset, the type and number of samples is shown.
