# Supplementary material for: LncRNA UCA1, Upregulated in CRC Biopsies and Downregulated in Serum Exosomes, Controls mRNA Expression by RNA-RNA Interactions
Source: Mol Ther Nucleic Acids. 2018 Jun 2;12:229–41. doi: 10.1016/j.omtn.2018.05.009 (PMC6023947; doi:10.1016/j.omtn.2018.05.009)
Supplement: Document S2. Article plus Supplemental Information [file mmc4.pdf]

Molecular Therapy
Nucleic Acids
Original Article

LncRNA UCA1, Upregulated in CRC Biopsies and Downregulated in Serum Exosomes, Controls mRNA Expression by RNA-RNA Interactions

Cristina Barbagallo,[1] Duilia Brex,[1] Angela Caponnetto,[1] Matilde Cirnigliaro,[1] Marina Scalia,[1] Antonio Magnano,[2] Rosario Caltabiano,[3] Davide Barbagallo,[1] Antonio Biondi,[4,6] Alessandro Cappellani,[4,6] Francesco Basile,[4,6] Cinzia Di Pietro,[1,6] Michele Purrello,[1,6] and Marco Ragusa[1,5,6]

[1]Department of Biomedical and Biotechnological Sciences, Section of Biology and Genetics G. Sichel, University of Catania, Catania 95123, Italy; [2]Digestive Endoscopy Service, Vittorio Emanuele Hospital, Catania 95124, Italy; [3]Department of Medical and Surgical Sciences and Advanced Technologies G.F. Ingrassia, University of Catania, Catania 95123, Italy; [4]Department of Surgery, Vittorio Emanuele Hospital, University of Catania, Catania 95124, Italy; [5]IRCCS Associazione Oasi Maria S.S., Institute for Research on Mental Retardation and Brain Aging, Troina (EN) 94018, Italy

Long non-coding RNAs (lncRNAs) and circular RNAs (circRNAs) contribute to the onset of many neoplasias through RNA-RNA competitive interactions; in addition, they could be secreted by cancer cells into biological fluids, suggesting their potential diagnostic application. By analyzing the expression of 17 lncRNAs and 31 circRNAs in biopsies and serum exosomes from colorectal cancer (CRC) patients through qRT-PCR, we detected CCAT1, CCAT2, HOTAIR, and UCA1 upregulation and CDR1AS, MALAT1, and TUG1 downregulation in biopsies. In serum exosomes, UCA1 was downregulated, while circHIPK3 and TUG1 were upregulated. Combined receiver operating characteristic (ROC) curves of TUG1:UCA1 and circHIPK3:UCA1 showed high values of sensitivity and specificity. Through *in vitro* (i.e., RNA silencing and mitogen-activated protein kinase [MAPK] inhibition) and *in silico* analyses (i.e., expression correlation and RNA-RNA-binding prediction), we found that UCA1 could (1) be controlled by MAPKs through CEBPB; (2) sequester miR-135a, miR-143, miR-214, and miR-1271, protecting ANLN, BIRC5, IPO7, KIF2A, and KIF23 from microRNA (miRNA)-induced degradation; and (3) interact with mRNA 3′-UTRs, preventing miRNA binding. UCA1 and its co-regulated antisense LINC01764 could interact and reciprocally mask their own miRNA-binding sites. Functional enrichment analysis of the RNA-RNA network controlled by UCA1 suggested its potential involvement in cellular migration. The UCA1 regulatory axis would represent a promising target to develop innovative RNA-based therapeutics against CRC.

INTRODUCTION

Non-coding RNAs (ncRNAs) are a highly heterogeneous group of untranslated RNA molecules having regulatory functions within eukaryotic cells. Based on their size, ncRNAs can be classified as (1) long non-coding RNAs (lncRNAs), longer than 200 nt; and (2) small non-coding RNAs, including microRNAs (miRNAs), whose length may be 200 nt or less.[1] LncRNAs regulate a plethora of fundamental biological processes (i.e., cell cycle, chromatin remodeling, histone modifications, splicing, and apoptosis) at different levels (transcriptional, post-transcriptional, translational, post-translational, and epigenetic).[2] However, the exact molecular mechanisms of action have been elucidated just for a few of them.

A particular class of lncRNAs is represented by circular RNAs (circRNAs): these are covalently closed RNA molecules produced from protein-coding genes through a splicing-like process, called backsplicing, during which the free ends of the transcript are bound in the typical head-to-tail junction.[3,4] The molecular mechanisms of action of lncRNAs and circRNAs are under intense investigation, but evidence suggests their involvement in a complex competitive endogenous RNA (ceRNA) network based on RNA-RNA interactions, including those between ncRNAs and mRNAs.[5,6] Indeed, lncRNAs and circRNAs may act as *miRNA sponges*: that is, they can bind and sequester miRNAs through miRNA-binding sites included within their sequences.[4,7] Moreover, lncRNAs could directly bind the 3′-UTR of mRNAs, protecting them against miRNA-induced degradation.[8]

In recent years, the discovery of ncRNAs has led to the investigation of their involvement in physiological and pathological processes. Moreover, ncRNAs have also been detected in biological fluids, often packaged within membrane-bound vesicles, such as exosomes.[9–12] Exosomes are small membrane vesicles characteristically cup shaped, with a diameter of 30–100 nm, containing proteins and RNAs (mRNAs, miRNAs, and lncRNAs);[13,14] recently, the presence of

Received 26 February 2018; accepted 11 May 2018;
https://doi.org/10.1016/j.omtn.2018.05.009.

[6]These authors contributed equally to this work.

Correspondence: Marco Ragusa, Department of Biomedical and Biotechnological Sciences, Section of Biology and Genetics G. Sichel, University of Catania, via Santa Sofia 87, 95123 Catania, Italy.
E-mail: mragusa@unict.it

This is an open access article under the CC BY license (http://creativecommons.org/licenses/by/4.0/).

Table 1. lncRNA and circRNA Expression in CRC Biopsies Compared to Normal Adjacent Tissues and in Serum Exosomes of CRC Patients Compared to Healthy Individuals

	Biopsies	Exosomes
CCAT1	8.37 (0.0007)[a]	NA
CCAT2	4.67 (0.001)[a]	−1.13 (0.38)
CDR1AS	−3.3 (0.002)[a]	NA
circCAMSAP1	−1.09 (0.46)	−1.39 (0.42)
circGON4L	−1.13 (0.28)	NA
circHIPK3	NA	3.82 (0.036)[a]
circLRP6	NA	1.45 (0.21)
circSPECC1	−1.12 (0.31)	NA
circZKSCAN1	1.19 (0.23)	NA
circZRANB1	1.07 (0.29)	NA
CRNDE	1.23 (0.37)	−1.37 (0.45)
H19	−1.41 (0.75)	NA
HOTAIR	9.17 (0.0008)[a]	1.42 (0.16)
LIT1	NA	−1.15 (0.69)
MALAT1	−1.8 (0.004)[a]	1.41 (0.35)
MEG3	−1.73 (0.93)	NA
MR17HG	1.32 (0.63)	NA
PCAT1	−1.61 (0.76)	−1.18 (0.31)
PCAT6	1.44 (0.81)	−1.19 (0.57)
PTENP1	1.31 (0.83)	NA
TUG1	−2.04 (0.036)[a]	1.69 (0.029)[a]
UCA1	7.14 (0.001)[a]	−4.22 (0.03)[a]
WRAP53	−1.26 (0.83)	NA
ZEB2AS1	1.14 (0.27)	−1.36 (0.95)

The t test p-value (in parentheses) and median fold change are shown for each ncRNA.
NA, not analyzed.
[a]Statistically significant.

double-stranded genomic DNA within circulating exosomes has been reported.[15] Exosomes are secreted by several, if not all, cell types, and they have been detected in all biological fluids analyzed to date.[16] Their main function is to mediate cell-to-cell communication through their molecular cargo, both in physiological and pathological conditions.[17] Several studies have reported a differential expression of ncRNAs within serum exosomes of patients affected by different diseases, including tumors; this has suggested the application of circulating ncRNAs for diagnostic and prognostic approaches.[18]

Colorectal cancer (CRC) is one of the most common diseases in developed countries, and it represents one of the tumors showing the most relevant dysregulation of ncRNA functions.[1,19–22] In the present study, we analyzed the expression of lncRNAs and circRNAs of oncological interest in tissues and serum exosomes from CRC patients, aiming to (1) increase our knowledge of the molecular bases of CRC, and (2) identify potential new biomarkers for its diagnosis and prognosis. This analysis led us to focus our attention on the lncRNA UCA1: through a combination of *in vitro* analyses (i.e., RNA silencing and inhibition of the mitogen-activated protein kinase [MAPK] pathway) and *in silico* approaches (i.e., expression correlation analysis and prediction of RNA-RNA binding), we reconstructed the ceRNA network controlled by UCA1 to get further insights into its molecular signaling.

RESULTS

NcRNA Expression in CRC Cell Lines and Exosomes

To verify their potential synthesis in CRC biopsies, the expression of selected lncRNAs and circRNAs was investigated through Real-Time PCR in two CRC cell lines: HCT-116 and Caco-2. The results of this preliminary screening showed that almost all analyzed molecules are expressed in both cell lines (Figure S1A). We performed the same analysis on exosomes secreted by the same cells, with the aim of evaluating the presence of lncRNAs and circRNAs within these vesicles. Except for circZRAMB1, all tested molecules were detected within the exosomes secreted by both CRC cell lines, even if at very low expression levels. In Figure S1B, we show the expression of exosomal ncRNAs characterized by a Ct value <35 in at least one cell line.

NcRNA Expression in FFPE CRC Biopsies and Serum Exosomes

To pinpoint the ncRNA molecules to be assayed in the entire cohort of patients, we performed a trial experiment on 3 formalin-fixed, paraffin-embedded (FFPE) biopsy pairs and serum exosomes compared to 3 healthy individuals (data not shown). Based on their expression (Ct < 35), we selected for FFPE biopsy analysis 15 of 17 lncRNAs (CCAT1, CCAT2, CRNDE, H19, HOTAIR, MALAT1, MEG3, MIR17HG, PCAT1, PCAT6, PTENP1, TUG1, UCA1, WRAP53, and ZEB2AS1) and 6 of 31 circRNAs (CDR1AS, circCAMSAP1, circGON4L, circSPECC1, circZKSCAN1, and circZRAMB1). Real-Time PCR analysis on 20 FFPE CRC biopsies and their normal adjacent tissues (NATs) showed significant differential expression of 6 lncRNAs and 1 circRNA (Table 1). More specifically, 4 lncRNAs (CCAT1, CCAT2, HOTAIR, and UCA1) were upregulated, while 2 lncRNAs (MALAT1 and TUG1) and 1 circRNA (CDR1AS) were downregulated in CRC tumor tissues compared to NATs.

Similarly, we selected for exosome analysis 10 lncRNAs (CCAT2, CRNDE, HOTAIR, LIT1, MALAT1, PCAT1, PCAT6, TUG1, UCA1, and ZEB2AS1) and 3 circRNAs (circCAMSAP1, circHIPK3, and circLRP6). Real-Time PCR analysis showed that 2 lncRNAs and 1 circRNA were differentially expressed (DE) in serum exosomes of CRC patients compared to healthy individuals: in particular, UCA1 was downregulated, while circHIPK3 and TUG1 were upregulated (Table 1) (Figure 1A). We computed Pearson's correlation coefficient (r) to evaluate the correlation of expression between DE ncRNAs in serum exosomes of all CRC patients and healthy individuals and in serum exosomes of CRC patients only. The Pearson coefficients showed a significant negative correlation between TUG1 and UCA1 in the entire cohort (Pearson = −0.45, p-value = 0.005), stronger in the CRC patient group (Pearson = −0.65, p-value = 0.004) (Figures 1B and 1C). Such a statistical correlation was not found in CRC

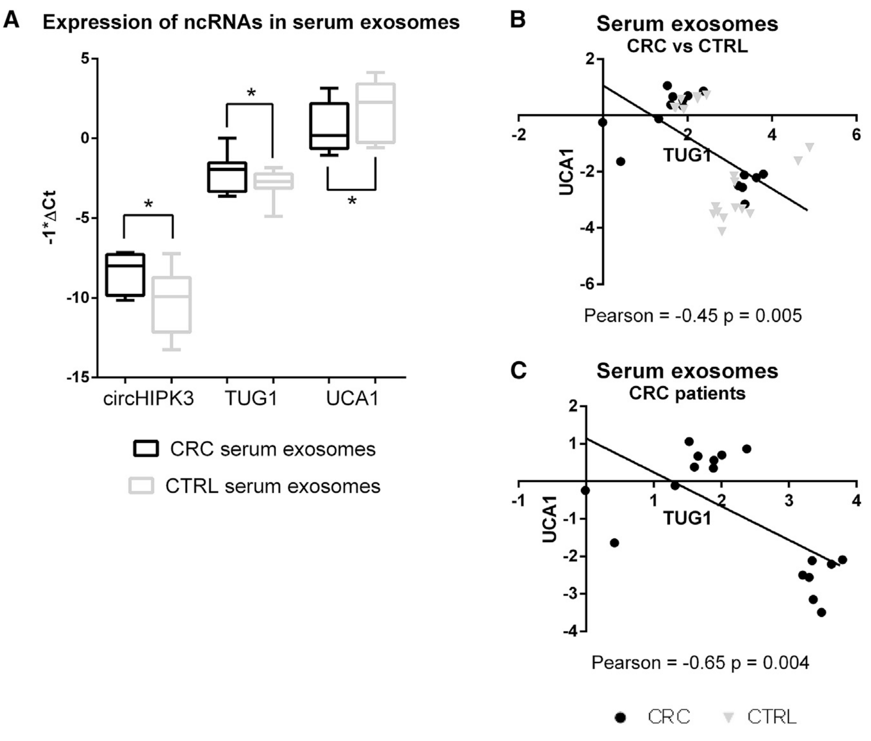

Figure 1. Boxplots Showing Differential Expression of ncRNAs in CRC Biopsies and Serum Exosomes

(A) DE ncRNAs in serum exosomes of CRC patients compared to healthy individuals. (B) Pearson's correlation coefficient (r) showed a negative correlation between TUG1 and UCA1 expression in serum exosomes of CRC patients and healthy individuals (C) and in serum exosomes of CRC patients only (D). *p ≤ 0.05 and **p ≤ 0.005.

tissues. No statistical correlation was observed between UCA1 and circHIPK3 and between TUG1 and circHIPK3.

Evaluation of Diagnostic Accuracy of DE ncRNAs in Serum Exosomes

We computed receiver operating characteristic (ROC) curves of DE ncRNAs using ΔCt values with respect to the endogenous control GAPDH. The circHIPK3 ROC curve showed an area under the curve (AUC) of 0.771 (95% confidence intervals [CIs], 0.508–0.936; p = 0.02) with 71% sensitivity and 80% specificity (ΔCt cutoff value ≤ 8.881197) (Figure 2A); the UCA1 curve showed an AUC of 0.719 (95% CIs, 0.533–0.863; p = 0.01) with 100% sensitivity and 43% specificity (ΔCt cutoff value ≤ 3.148002) (Figure 2B); the TUG1 curve was not significant (p = 0.2). The TUG1:UCA1 combined ROC curve, designed using ΔCt values calculated as $Ct_{TUG1} - Ct_{UCA1}$, showed an AUC of 0.814 (95% CIs, 0.627–0.933; p = 0.0001) with 93% sensitivity and 64% specificity (ΔCt cutoff value ≤ 5.855948) (Figure 2C). The circHIPK3:UCA1 combined ROC curve, computed using ΔCt values calculated as $Ct_{circHIPK3} - Ct_{UCA1}$, showed an AUC of 0.9 (95% CIs, 0.648–0.992; p < 0.0001) with 100% sensitivity and 70% specificity (ΔCt cutoff value ≤ 12.651507) (Figure 2D).

Analysis of GEO Datasets Confirms the Deregulation of DE ncRNAs

To validate the differential expression of ncRNAs observed in CRC FFPE biopsies and serum exosomes, we reanalyzed the expression of these ncRNAs in 15 CRC GEO datasets. The ncRNA expression data extracted from GEO also confirmed our data in larger cohorts of CRC tissues and normal colon mucosa. This analysis revealed a positive correlation between the up- or downregulation of each DE ncRNA and CRC progression and clinical and genetic features (such as tumor stage and size, relapse, metastasis, and CRC-associated mutations) (Figures 3A–3D). In addition, a dataset on peripheral blood of CRC patients compared to healthy individuals was analyzed, confirming the upregulation of TUG1 and the downregulation of UCA1 observed in our cohort (Figure 3E). Specifically, the same deregulation trend of both lncRNAs was observed in two different groups of CRC patients, divided according to Dukes classification: (1) Dukes stages A and B, and (2) Dukes stages C and D. In the same dataset, the negative correlation of expression between TUG1 and UCA1 (here described in serum exosomes) was confirmed (Pearson = −0.43, p-value = 0.01) (Figure 3F). Dataset screening provided no results for CCAT1, CCAT2, CDR1AS, and circHIPK3, because no specific probes for these ncRNAs were present in the analyzed datasets.

Inhibition of MAPKs Affects ncRNA Expression

As the MAPK pathway plays a master control role in various CRC-related biological processes, we evaluated the effects of MAPK inhibition on the expression of ncRNAs showing altered expression in CRC in our study (Figures S2A and S2B). The expression of ncRNAs was investigated in HCT-116 cells at 6, 12, 24, and 48 hr after treatment with U0126. The results showed that the expression of DE ncRNAs was affected by the inhibition of the MAPK pathway (Table 2). More specifically, all ncRNAs, excluding HOTAIR and TUG1, significantly decreased their expression at 6 hr, while their expression levels increased at 24 hr, except for CCAT1, which was consistently downregulated at 6, 12, and 24 hr but upregulated at 48 hr.

To corroborate our findings on other experimental CRC models, we investigated within GEO datasets the relationship between the expression of post-treatment deregulated ncRNAs and the activation of the MAPK pathway. More precisely, we computed the expression correlation (Pearson coefficient) between each DE ncRNA and HSPA5, a transcriptional target of phospho-Erk (p-Erk).[23] According to our data, we expected a positive correlation between HSPA5 and ncRNAs

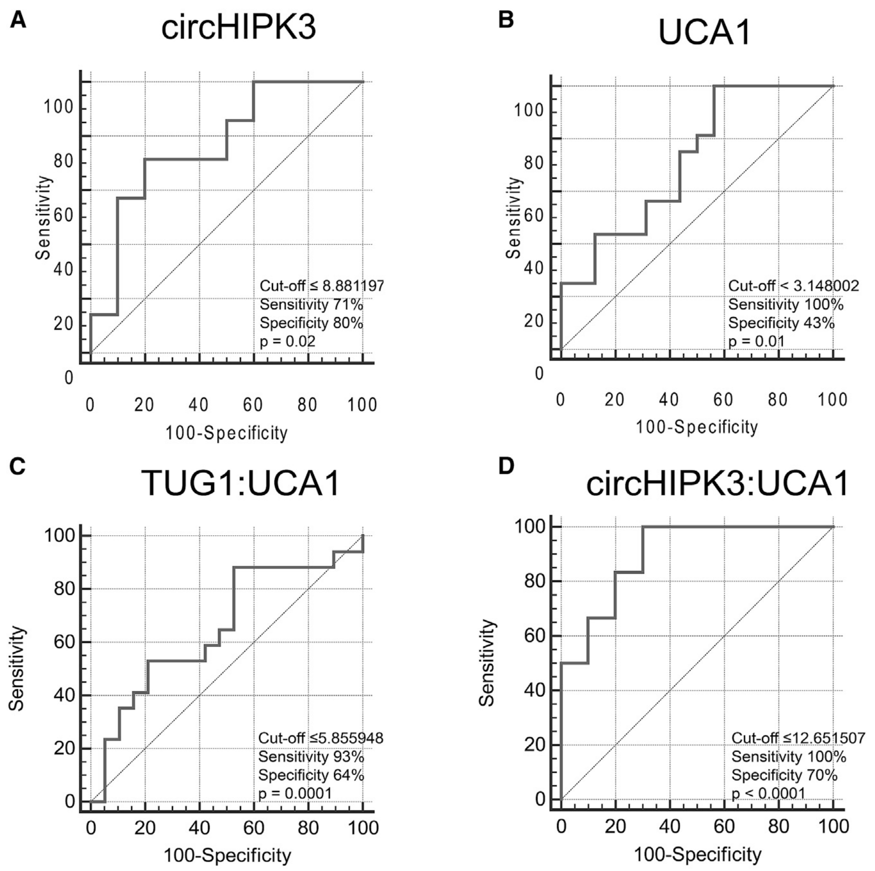

Figure 2. ROC Curves of DE ncRNAs in Serum Exosomes of CRC Patients Compared to Healthy Individuals

ROC curves showing the sensitivity and specificity as CRC biomarkers of (A) circHIPK3 and (B) UCA1. (C) Combined ROC curve of TUG:UCA1 ($Ct_{TUG1} - Ct_{UCA1}$). (D) Combined ROC curve of circHIPK3:UCA1 ($Ct_{circHIPK3} - Ct_{UCA1}$).

showing decreased expression at 6 hr post-treatment. Results showed both positive and negative correlations in different datasets, but UCA1 data were more consistent with a positive linear correlation with HSPA5, while MALAT1 showed high heterogeneity (Table S1). These data would hint to a hypothetical control of UCA1 expression induced by the activation of MAPK signaling.

Identification of Regulation Axes of UCA1

Because of its asymmetric deregulation between tissues and serum exosomes and its potential association with the MAPK pathway, we focused our subsequent *in silico* and *in vitro* analyses on UCA1 to get a better insight into its biomolecular roles and related regulatory circuits. We overlapped different tracks from the University of California, Santa Cruz (UCSC) Genome Browser, which had been derived from Encyclopedia of DNA Elements (ENCODE) experiments. This allowed us to identify the transcription factor-binding sites (TFBSs) for CEBPB, TEAD4, TFAP2A, and TFAP2C within 1 kb upstream of the UCA1 transcription start site. The expression of these TFs in CRC tumor tissues and their correlation of expression with UCA1 were investigated in GEO and The Cancer Genome Atlas (TCGA) datasets. CEBPB and TEAD4 showed the most significant upregulation and positive correlation with UCA1 (Table 3; Table S2). The expression of CEBPB and TEAD4 was also investigated after MAPK inhibition, showing a decrease for both TFs at 6 and 12 hr after treatment and an increase at 24 and 48 hr (Table 3). Importantly, the expression trends of CEBPB and UCA1 showed a significant linear correlation (Spearman = 0.66, p value = 0.0004), suggesting that CEBPB may be involved in UCA1 transcription regulation.

To investigate the RNA-based signaling of UCA1, we hypothesized that UCA1 could regulate mRNA decay by sequestering miRNAs, thus preventing them from repressing their mRNA targets. Accordingly, we expected that in the module UCA1:miRNAs:mRNAs, UCA1 and mRNAs would show a significant co-expression. Based on sequence complementarity between miRNA seed regions and the lncRNA sequence, the miRcode tool allowed us to identify miRNAs that could be sponged by UCA1. Among them, we selected as hypothetical targets of UCA1 4 miRNAs (miR-135a, miR-143, miR-214, and miR-1271), showing from 1 to 4 binding sites within the UCA1 sequence and reduced expression in CRC according to GEO dataset screening (Table S3). Experimentally validated targets of the 4 miRNAs were retrieved from StarBase, miRTarbase, and the literature (Table S4), while their expression in CRC was assessed by Oncomine and analyzed in GEO and TCGA expression datasets. In Table S5, we list the 14 miRNA targets selected, which are characterized by upregulation in CRC and have positive correlation with UCA1.

The expression of miR-135a, miR-143, miR-214, and miR-1271 was investigated after inhibition of the MAPK pathway: a statistically significant upregulation of miR-143 was observed at 12 and 24 hr after treatment, while miR-214 showed the same deregulation trend at 48 hr after treatment (Table 3). Similarly, we assayed the expression of mRNA targets of miR-143 and miR-214 after the same treatment, showing significant alterations of ANLN, BIRC5, CD46, DEK, DNMT3A, KIF23, HMMR, and MACC1 at different time points (Table 3). A significant positive correlation was shown between expression levels of UCA1 and BIRC5 (Spearman = 0.39, p-value = 0.05), CD46 (Spearman = 0.79, p-value < 0.0001), DNMT3A (Spearman = 0.75, p-value < 0.0001), HMMR (Spearman = 0.4, p-value = 0.05), and MACC1 (Spearman = 0.43, p-value = 0.03). Although IPO7 expression was not significantly altered by U0126

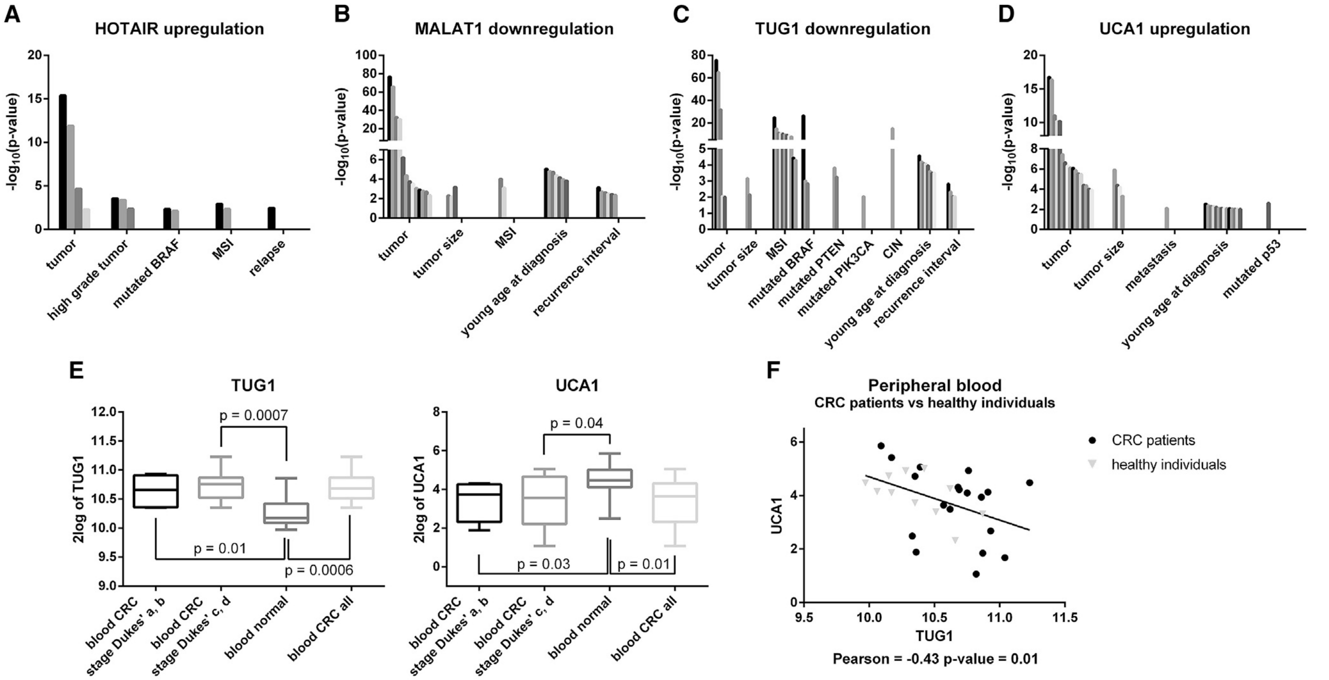

Figure 3. Validation of ncRNA Dysregulation in Tissues and Peripheral Blood of CRC Patients by GEO Dataset Screening

(A–D) GEO dataset analysis confirmed the upregulation of (A) HOTAIR and (B) UCA1 and the downregulation of (C) MALAT1 and (D) TUG1 in CRC tissues compared to normal colon mucosa. This analysis revealed a statistically significant positive association between ncRNA up- or downregulation and CRC progression and clinical and genetic features. Statistical significance is shown as $-\log_{10}$ (p value). Each dataset is represented by a different bar. MSI, microsatellite instability; CIN, chromosomal instability. (E) The dataset GEO: GSE10715 confirmed the upregulation of TUG1 and the downregulation of UCA1. The same trend is observed in two different groups of CRC patients, composed of (1) Dukes stages A and B, and (2) Dukes stages C and D. (F) Significant negative correlation of expression between TUG1 and UCA1.

treatment, it was strongly positively correlated to UCA1 expression (Spearman = 0.76, p-value < 0.0001). These data would suggest that expression correlation between UCA1 and some of its potential targets persisted also after the *in vitro* perturbation induced by U0126 treatment. Despite the fact that miR-135a expression was not altered by U0126 treatment, we investigated the expression of MYC, which was selected as a target of miR-135a. It has been reported that MYC expression is promoted by CCAT1 and CCAT2,[24,25] whose expression was altered by MAPK inhibition. MYC showed a significant downregulation at 6 and 12 hr post-treatment, similar to the expression trend of CCAT1 and CCAT2 (Table 3). Moreover, MYC and CCAT2 showed a significant positive correlation of expression (Spearman = 0.89, p-value < 0.0001).

We found that both long and short variants of LINC01764, a noncoding antisense on the UCA1 locus, include a region longer than 100 nt showing sequence complementarity on the 3′-UTR of UCA1. More specifically, the LINC01764 short variant would bind the UCA1 region harboring the miR-214-binding site, while the complementary region between UCA1 and the LINC01764 long variant overlapped the binding sites of miR-135a and miR-143 on the LINC01764 sequence (Figure 4A). The expression of LINC01764 after MAPK inhibition was decreased at 6 and 12 hr after treatment and significantly increased at 24 and 48 hr (Table 3). Moreover, LINC01764 levels were parallel to the expression of UCA1 during the same time course (Spearman = 0.42, p-value = 0.03). These data would suggest a potential physical interaction between UCA1 and LINC01764, which could reciprocally mask their own miRNA-binding sites and, accordingly, explain the expression correlation observed between UCA1 and its antisense.

UCA1 Target Expression after *In Vitro* Silencing

The miRNAs potentially sponged by UCA1 were moderately upregulated at 24 hr post-transfection (PT) with ASOs (antisense oligonucleotides). In particular, miR-214 and miR-1271 showed the highest fold change values at 24 hr PT (Table 4). Moreover, UCA1 expression levels showed a significant negative correlation with miR-135a (Spearman = −0.8, p-value = 0.01) and miR-143 (Spearman = −0.52, p-value = 0.02). In the same way, expression analysis was also performed on experimentally validated miRNA targets: ANLN, BIRC5, IPO7, KIF2A, and KIF23 showed a statistically significant downregulation at 48 hr PT (Table 4). These results would show a potential positive control of UCA1 on ANLN, BIRC5, IPO7, KIF2A, and KIF23 expression. Although LINC01764 was downregulated at 24 and 48 hr after UCA1 silencing, its variation was not statistically significant, but it did show a significant positive correlation with UCA1 expression (Spearman = 0.47, p-value = 0.04).

Table 2. ncRNA Expression after U0126 Treatment

ncRNA	6 hr	12 hr	24 hr	48 hr
CCAT1	−3.6 (0.013)[a]	−2.34 (0.026)[a]	−2.48 (0.022)[a]	2.32 (0.015)[a]
CCAT2	−3.56 (0.05)[a]	−1.15 (0.39)	2.33 (0.001)[a]	2.5 (0.015)[a]
CDR1AS	−9.88 (0.002)[a]	−1.29 (0.24)	4.88 (0.004)[a]	−1.02 (0.36)
HOTAIR	−1.89 (0.18)	1.23 (0.53)	3.27 (0.01)[a]	2.8 (0.002)[a]
circHIPK3	−3.13 (0.023)[a]	1.18 (0.41)	2.26 (0.005)[a]	1.95 (0.033)[a]
MALAT1	−3.69 (0.017)[a]	1.43 (0.2)	2.61 (0.001)[a]	1.36 (0.13)
TUG1	−1.13 (0.068)	1.31 (0.45)	2.38 (0.005)[a]	2.77 (0.0004)[a]
UCA1	−4.29 (0.016)[a]	1.58 (0.12)	2.36 (0.006)[a]	2.14 (0.003)[a]

Median fold change and t test p-value (in parentheses) are shown.
[a]Statistically significant.

Prediction of Direct Binding between UCA1 and the 3′-UTR of ANLN, BIRC5, IPO7, KIF2A, and KIF23

Additionally, we tested the hypothesis that mRNA downregulation induced by UCA1 silencing could be the effect of its direct binding on mRNA 3′-UTRs, which would protect them from miRNA binding and, thus, increase mRNA stability. Using the IntaRNA tool, we computed the potential RNA-RNA interactions among UCA1 and the 3′-UTRs of mRNAs downregulated PT. We found that binding of UCA1 on the 3′-UTRs of mRNAs showed several regions with strong negative free energy (NFE) (i.e., index of very effective binding), which overlapped several miRNA-binding sites identified by the microrna.org database (Figure 4B; Table S6). More specifically, we detected 17, 5, 16, 10, and 10 miRNAs whose binding sites lay in such NFE regions for ANLN, BIRC5, IPO7, KIF2A, and KIF23, respectively. About 80% of these 44 miRNAs exert a tumor suppressor role in CRC, according to GEO datasets (Table S7). These findings could suggest that UCA1, by binding the 3′-UTRs of mRNAs, would mask binding sites of miRNAs with a potential tumor-suppressive role in CRC, thus decreasing miRNA-induced degradation of mRNAs.

Functional Enrichment Analysis of the ceRNA Network Controlled by UCA1

To evaluate the biological functions potentially regulated by the ceRNA network controlled by UCA1, we computationally created a network of molecular interactions among Anln, Birc5, Ipo7, Kif2a, and Kif23 proteins and their first interactors. We performed functional enrichment analysis on all network nodes, showing a statistical over-representation of pathways associated with CRC carcinogenesis (e.g., MAPK, TGF-β, and Wnt pathways) or regulating cancer-related processes, such as cell cycle, apoptosis, adhesion, and cytoskeleton organization and migration; interestingly, processes related to mRNA stability and processing were also over-represented (Figure S3; Table S8).

DISCUSSION

NcRNA Dysregulation Is Associated with the CRC Phenotype

Our data show that 6 lncRNAs (CCAT1, CCAT2, HOTAIR, MALAT1, TUG1, and UCA1) and 1 circRNA (CDR1AS) are differentially expressed in tumor tissues compared to normal mucosa; in particular, we observed the upregulation of CCAT1, CCAT2, HOTAIR, and UCA1 and the downregulation of CDR1AS, MALAT1, and TUG1. We confirmed these data by statistical re-analysis of expression microarray datasets from large cohorts of CRC patients with probes specific for ncRNAs. Interestingly, upregulation of both HOTAIR and UCA1 was statistically associated with several worse prognostic factors (e.g., high-grade tumors, tumor size, microsatellite instability, and BRAF and P53 mutations). Similarly, downregulation of MALAT1 and TUG1 was related to tumor size, microsatellite instability, and young age at diagnosis and recurrence interval. These data strongly suggest that the alteration of these lncRNAs could contribute to the progression of CRC.

High HOTAIR and UCA1 levels had been previously observed in several tumors, including CRC. HOTAIR upregulation has also shown a positive correlation with poor prognosis, epithelial-mesenchymal transition (EMT), and metastasis in CRC patients.[26–28] UCA1 expression is increased in tumor tissues of patients with lymph node metastasis compared to non-metastatic ones,[29] and its upregulation correlates with tumor stage, poor prognosis, and metastasis.[30] Although the downregulation of MALAT1 and TUG1 observed here was in agreement with their decreased expression in several other tumors,[31–35] it differed from other reports about CRC, in which the upregulation of MALAT1 and TUG1 was related to poor prognosis and metastasis.[36–39] It is noteworthy that the published data report a very complex scenario about MALAT1 and TUG1 expression in different tumors, and, in some cases, opposite functions have been reported in the same cancer model.[33,35,40,41] It is possible that the molecular functions involving lncRNAs are strongly dependent on the specific molecular context in which lncRNAs act, which is highly heterogeneous also within the same cancer type. The expression dysregulation reported here for CCAT1, CCAT2, and CDR1AS has not been supported by statistical re-analysis of CRC microarray datasets because no specific probe for these ncRNAs was available. Previous studies reported the upregulation of CCAT1 and CCAT2 in CRC tissues and their involvement in proliferation, invasion, and migration;[25,42–44] a recent paper reported the upregulation of CDR1AS in CRC tissues compared to normal colon mucosa.[45]

Interestingly, the expression of all DE ncRNAs analyzed was modified by drug-induced inhibition of the MAPK pathway, whose hyperactivation is considered a key event for CRC onset and progression.[46,47] It is well known that the molecular effects of U0126 appear a few hours after treatment; accordingly, we observed a global downregulation all ncRNAs at 6 hr after treatment, except for HOTAIR and TUG1. On the other hand, ncRNAs showed a global upregulation at 24 and 48 hr after treatment (when the drug effect had likely terminated), which could represent a compensation effect due to pathway reactivation. The early down-modulation of ncRNAs after MAPK inhibition partially matched the positive expression correlation between HSPA5 (transcriptional target of p-Erk) and MALAT1 and UCA1. These data would suggest a potential transcriptional control of these ncRNAs from MAPK signaling, which seems more consistent for UCA1.

Table 3. Expression of TFs, miRNAs, mRNAs, and LINC01764 after U0126 Treatment

miRNAs Targeting mRNAs Co-expressed with UCA1	mRNAs/miRNAs	6 hr	12 hr	24 hr	48 hr
–	miR-135a	1.38 (0.24)	−1.03 (0.82)	1.12 (0.73)	−1.73 (0.14)
–	miR-143	1.29 (0.15)	1.5 (0.028)[a]	1.56 (0.027)[a]	1.16 (0.43)
–	miR-214	−1.94 (0.88)	−2.34 (0.23)	1.13 (0.61)	3.14 (0.02)[a]
–	miR-1271	1.23 (0.21)	1.03 (0.71)	1.31 (0.91)	−1.36 (0.14)
miR-135a, miR-214, miR-1271	ANLN	−1.38 (0.41)	−1.02 (0.6)	−1.21 (0.26)	−4.19 (0.039)[a]
miR-135a, miR-143	BIRC5	−4.19 (0.032)[a]	1.23 (0.61)	1.74 (0.23)	4.57 (0.008)[a]
miR-135a, miR-143, miR-214	CD46	1.55 (0.23)	−1.76 (0.05)[a]	2.13 (0.014)[a]	1.27 (0.07)
miR-135a, miR-214	DEK	−4.3 (0.014)[a]	−1.15 (0.5)	−1.31 (0.18)	−1.26 (0.2)
miR-143	DNMT3A	−1.87 (0.11)	1.41 (0.75)	1.92 (0.009)[a]	1.15 (0.72)
miR-143, miR-1271	HMMR	−2.13 (0.17)	−1.01 (0.59)	1.34 (0.005)[a]	−2.62 (0.07)
miR-214	IPO7	−1.13 (0.41)	−1.35 (0.25)	1.77 (0.09)	1.08 (0.84)
miR-135a, miR-143, miR-1271	KIF23	−1.87 (0.19)	−1.25 (0.28)	1.2 (0.17)	−2.53 (0.05)[a]
–	LINC01764	−2.65 (0.08)	−1.22 (0.23)	2.83 (0.004)[a]	4.8 (0.008)[a]
miR-143	MACC1	−3.11 (0.05)[a]	1.4 (0.034)[a]	2.55 (0.0006)[a]	1.54 (0.06)
miR-143	MMP7	5.01 (0.25)	2.37 (0.06)	2.2 (0.09)	1.19 (0.83)
miR-135a	MYC	−5.29 (0.013)[a]	−4.98 (0.023)[a]	1.25 (0.08)	2.21 (0.09)
–	CEBPB	−3.39 (0.01)[a]	−1.08 (0.55)	2.08 (0.001)[a]	1.72 (0.046)[a]
–	TEAD4	−1.99 (0.049)[a]	−1.28 (0.21)	3.43 (0.002)[a]	1.64 (0.07)

Median fold change and t test p-value (in parentheses) are shown.
[a]Statistically significant.

Indeed, very reliable TFBSs for CEBPB and TEAD4 were found on the upstream regulatory region of UCA1. Dysregulation of CEBPB and TEAD4 during U0126 treatment was analogous to the UCA1 deregulation trend; moreover, expression of CEBPB and UCA1 showed a strong significant linear correlation. This would suggest that CEBPB may act as a transcription activator for UCA1 through the MAPK pathway. This hypothesis is in agreement with several papers reporting that CEBPB harbors a highly conserved MAPK consensus site, whose phosphorylation operated by MAPKs regulates its activity.[48–50] Accordingly, CEBPB has been associated with several tumor models, including CRC, where it has shown increased expression compared to normal mucosa.[51,52]

NcRNAs in Serum Exosomes of CRC Patients May Be Applied in Non-invasive Diagnosis

Expression analysis of ncRNAs in serum exosomes of CRC patients showed the upregulation of circHIPK3 and TUG1 and the downregulation of UCA1. circHIPK3 expression has been investigated in several cancer cell lines, including CRC cells, showing higher levels compared to the linear HIPK3 transcripts.[53] Its presence in serum exosomes was previously reported,[54] but no association with CRC was observed. We describe for the first time the presence of TUG1 within exosomes from human serum and its deregulation in CRC patient serum. The possible application of circulating TUG1 as a diagnostic and prognostic biomarker has already been reported in other cancer models.[55,56] To date, UCA1 deregulation in serum exosomes of CRC patients has never been reported; on the contrary, its upregulation was observed in CRC patient plasma.[29] However, the analysis of the dataset GEO: GSE10715[57] allowed us to validate our results in an independent cohort of 19 CRC patients and 11 healthy individuals, confirming the upregulation of TUG1 and the downregulation of UCA1, as well as their negative expression correlation. Our data showed an opposite deregulation trend in CRC tissues and in serum exosomes for TUG1, which is downregulated within tumor cells and upregulated in serum exosomes. This observation may suggest that tumor cells protect themselves from TUG1 tumor-suppressive activity by secreting the lncRNA via exosomes. Similar to TUG1, UCA1 showed an asymmetric distribution between tumor tissues, where the lncRNA is upregulated, and serum exosomes, where it is downregulated. This opposite deregulation would suggest that the oncogenic function of UCA1 is crucial for tumor progression, inducing tumor cells to retain the lncRNA by limiting its secretion through exosomes.[58]

ROC curves showed good accuracy for 2 of 3 DE ncRNAs (circHIPK3: 71% sensitivity, 80% specificity; UCA1: 100% sensitivity, 43% specificity), with significant p-values. On the contrary, the TUG1 ROC curve showed no statistical significance. The goal of using diagnostic and prognostic biomarkers is to detect a disease with the highest possible accuracy, and a single biomarker is often not sufficient. For this reason, the use of a signature of multiple biomarkers is preferable, as it would increase diagnostic and prognostic accuracy.[59] We thus computed combined ROC curves considering two ncRNAs with opposite trends of expression at a time (ΔCt values obtained as $Ct_{TUG1} - Ct_{UCA1}$ and as $Ct_{circHIPK3} - Ct_{UCA1}$ were used to compute

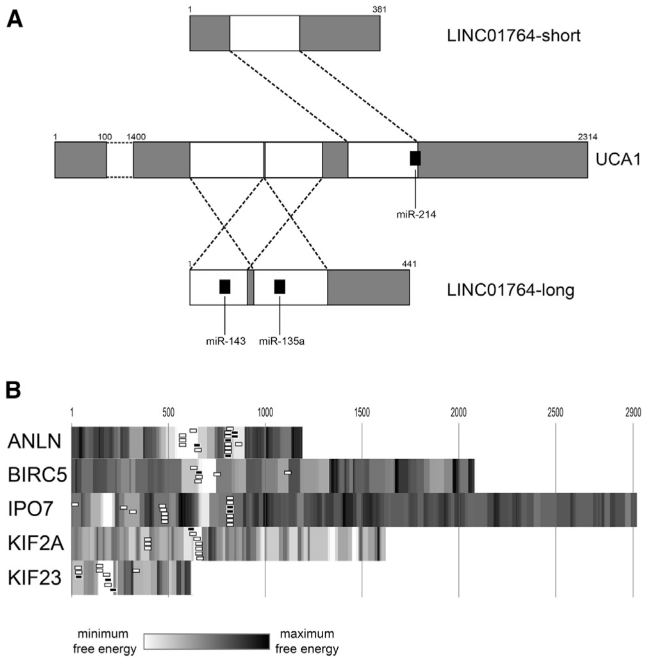

Figure 4. Prediction of UCA1 RNA Interactions

(A) Complementarity between UCA1 and the two variants of its antisense RNA (LINC01764). The white areas show the complementary regions between UCA1 3′-UTR and the long and short variants of LINC01764; the black boxes represent the miRNA-binding sites overlapping complementary regions. (B) Graphical representation of free energy (kJ) computed from the binding of UCA1 and the 3′-UTRs of its co-expressed mRNAs. Gray-coded scale, based on free energy values, is used to depict the 3′-UTRs of mRNAs. White regions depict the binding regions with minimum free energy, and, accordingly, they represent the most reliable binding regions. The boxes represent miRNAs binding to minimum free energy regions: white boxes, tumor suppressor miRNAs in CRC; black boxes, onco-miRNAs in CRC. Free energy scale limits: (1) ANLN: minimum = −22.8, maximum = −0.28; (2) BIRC5: minimum = −27.32, maximum = −2.2; (3) IPO7: minimum = −30.17, maximum = 0.82; (4) KIF2A: minimum = −15.11, maximum = 3.01; and (5) KIF23: minimum = −15.01, maximum = −0.38.

two different curves).[60,61] Both combined ROC curves were statistically significant, showing lower p-values, higher AUC values, and a better combination of sensitivity and specificity values than single ROC curves, thus demonstrating an increased diagnostic accuracy. These data support the hypothesis that the combination of two ncRNAs may diagnose CRC with higher sensitivity and specificity compared to a single biomarker. Further analyses on larger cohorts will be necessary to evaluate the diagnostic accuracy of these molecules as CRC biomarkers.

UCA1 Potentially Contributes to CRC Pathogenesis by Regulating an RNA Network

Several studies reported that UCA1 may act as an miRNA sponge in different *in vitro* models. It has been reported that enforced expression of UCA1 resulted in decreased expression of its miRNA targets, or, vice versa, reduced expression of UCA1 caused increased levels of miRNAs.[62–65] Based on this evidence, we investigated the effect of UCA1 silencing on its hypothetical miRNA targets, miR-135a, miR-214, miR-143, and miR-1271, observing a slight trend of upregulation for all miRNAs at 24 hr after transfection. We also analyzed the expression of experimentally validated mRNA targets of these miRNAs, selected because of their positive correlation of expression with UCA1.

Table 4. Expression of miRNAs, mRNAs, and LINC01764 after UCA1 Silencing

miRNAs Targeting mRNAs Co-expressed with UCA1	miRNAs/ mRNAs	24 hr	48 hr
–	miR-135a	1.26 (0.25)	1.24 (0.26)
–	miR-143	1.11 (0.56)	−1.15 (0.39)
–	miR-214	1.61 (0.3)	−1.12 (0.81)
–	miR-1271	1.76 (0.34)	−3 (0.27)
miR-135a, miR-214, miR-1271	ANLN	−1.23 (0.34)	−1.56 (0.003)[a]
miR-135a, miR-143	BIRC5	−1.21 (0.05)[a]	−1.43 (0.009)[a]
miR-135a	BZW2	1.01 (0.59)	−1.29 (0.072)
miR-135a, miR-143, miR-214	CD46	−1.19 (0.22)	−1.02 (0.75)
miR-135a, miR-214	DEK	−1.29 (0.3)	−1.09 (0.58)
miR-143	DNMT3A	−1.07 (0.19)	−1.34 (0.13)
miR-143, miR-1271	HMMR	−1.32 (0.21)	−1.12 (0.37)
miR-214	IPO7	−1.35 (0.19)	−1.72 (0.004)[a]
miR-1271	KIF2A	−1.32 (0.38)	−1.9 (0.008)[a]
miR-135a, miR-143, miR-1271	KIF23	−1.26 (0.076)	−1.74 (0.007)[a]
–	LINC01764	−1.49 (0.16)	−1.18 (0.58)
miR-143	MACC1	−1.21 (0.23)	−1.32 (0.18)
miR-143	MMP7	−1.22 (0.56)	1.34 (0.072)
miR-135a	MYC	1.08 (0.42)	1.24 (0.52)
miR-135a	TRIP13	−1.05 (0.27)	−1.2 (0.61)

Median fold change and t test p-value (in parentheses) are shown.
[a]Statistically significant.

Among miRNA targets, ANLN, IPO7, KIF2A, and KIF23 showed a significant reduced expression at 48 hr after transfection, while BIRC5 levels were decreased both at 24 and 48 hr. It is conceivable that miRNAs sponged by lncRNAs are no longer available for binding the 3′-UTR of mRNA targets, without alterations of their expression levels. This may explain why no statistically significant alterations were observed in miRNA expression after UCA1 silencing. According to the hypothesis of miRNA sponging, when UCA1 levels decrease, miRNAs are no longer sequestered and are free to bind their mRNA targets, which, in turn, show decreased expression. Indeed, the downregulation of ANLN, IPO7, KIF2A, and KIF23 was observed at 48 hr after UCA1 silencing, while BIRC5 levels were already decreased at 24 hr. Intriguingly, such downregulation could be due to a direct protection on mRNAs performed by UCA1, which could bind their 3′-UTR and prevent miRNA binding. Following this second hypothesis, we computationally found reliable interactions between UCA1 and the 3′-UTRs of ANLN, BIRC5, IPO7, KIF2A, and

KIF23 that overlapped several miRNA-binding sites of tumor-suppressor miRNAs.

Finally, we hypothesized that UCA1 and its antisense LINC01764 could be involved in a mechanism of reciprocal regulation of expression, due to a mutual protection from binding of miR-214 on UCA1 and miR-143 and miR-135a on the LINC01764 long isoform. Accordingly, LINC01764 and UCA1 showed a significant positive correlation of expression after U0126 treatment and UCA1 silencing. Taken together, this evidence would suggest the existence of a co-regulation mechanism of UCA1 and LINC01764 based on RNA interactions. The biological role of LINC01764 still remains unknown.

The UCA1 network showed strong over-representation of processes related to actin and cytoskeleton organization, which underlie the migration phenomenon. Accordingly, UCA1 was previously reported to induce migration in cancer.[66–68] Moreover, functional enrichment analysis showed the over-representation of processes regulating mRNA stability and processing. These results support our hypothesis, suggesting that UCA1 controls key biological processes promoting CRC progression by regulating the expression of ANLN, BIRC5, IPO7, KIF2A, and KIF23.

Conclusions

Based on our hypotheses, UCA1 may be an RNA regulator of CRC progression by controlling a ceRNA network, thus fostering the upregulation of ANLN, BIRC5, IPO7, KIF2A, and KIF23 in two ways: (1) by sponging miRNAs negatively regulating their expression, and (2) by directly binding the 3′-UTRs of mRNAs and protecting them from miRNA-mediated degradation. The existence of this complex RNA-based regulatory signaling, which controls cancer-related pathways, paves the way to innovative therapeutic applications of RNA-based anticancer strategies targeting UCA1.

MATERIALS AND METHODS

Cell Lines

HCT-116 and Caco-2 cell lines, both derived from primary tumors, were obtained from the Interlab Cell Line Collection (ICLC), an International Certified Repository Authority within the IRCCS Azienda Ospedaliera Universitaria San Martino-IST Istituto Nazionale per la Ricerca sul Cancro (Genova, Italy). HCT-116 cells were cultured in RPMI-1640 medium (Gibco, Thermo Fisher Scientific, Waltham, MA), supplemented with 10% fetal bovine serum (FBS) (Gibco) and 2 mM L-glutamine (Lonza, Basel, Switzerland); Caco-2 cells were cultured in DMEM (Sigma-Aldrich, St. Louis, MO), supplemented with 20% FBS (Gibco), 2 mM L-glutamine (Lonza), and 1% non-essential amino acids (NEAA) (Lonza); both media were supplemented with 1% penicillin/streptomycin (10,000 U/mL) (Gibco). Cells were cultured at 37°C and 5% CO_2.

Isolation of Exosomes Secreted by CRC Cell Lines

Exosomes secreted by both cell lines were isolated from culture media 3 days after seeding in 175-cm^2 flasks (6×10^6 cells/flask), as previously reported.[69] Briefly, medium was harvested and centrifuged at 300 $\times$ g (4°C, 10 min) on a Beckman J-6M/E centrifuge (rotor TY.JS 5.2) (Beckman Coulter, Brea, CA) to pellet debris. The supernatant was then centrifuged at 16,500 $\times$ g (4°C, 30 min) on a Beckman L8 70M ultracentrifuge (rotor SW28), and then filtered through a 0.2-μm filter. The final supernatant was centrifuged at 120,000 $\times$ g (4°C, 70 min) with the same ultracentrifuge. Exosome pellet was directly lysed for RNA isolation. FBS added to culture medium had been previously deprived of exosomes through the same protocol, removing the exosome pellet and harvesting the FBS supernatant for subsequent use.

RNA Isolation from CRC FFPE Samples and Serum Exosomes

20 CRC patients were recruited at Azienda Ospedaliero-Universitaria Policlinico Vittorio Emanuele (Catania, Italy). FFPE tumor tissues and distal normal colon mucosa were isolated at the Section of Anatomic Pathology, Department G.F. Ingrassia, University of Catania (Catania, Italy). RNA was extracted from FFPE samples through a PureLink FFPE RNA Isolation Kit (Invitrogen), according to the manufacturer's instructions. Serum of these 20 CRC patients and 20 unaffected individuals was collected through venous blood sampling after having signed informed consent for the use of their blood, in accordance with the Declaration of Helsinki. This study was approved by the Ethical Committee of "Vittorio Emanuele" Hospital (Azienda Ospedaliero-Universitaria Policlinico Vittorio Emanuele, Catania). To separate serum from cellular components, blood samples were centrifuged at 15,000 $\times$ g (4°C, 15 min), and then the supernatant was harvested and centrifuged again under the same conditions to completely remove cells or debris, as these could alter subsequent analysis. Supernatant serum was harvested and mixed to ExoQuick (System Biosciences, Palo Alto, CA) to precipitate nanovesicles, according to the manufacturer's instructions. Finally, the exosome pellet was directly lysed with TriZol for total RNA isolation. RNA was quantified by GenQuant pro spectrophotometer (Biochrom, Cambridge, UK) and Qubit (Invitrogen, Thermo Fisher Scientific, Waltham, MA), and treated with DNase (Invitrogen), according to the manufacturer's instructions. Notably, both procedures used for exosome isolation (ultracentrifugation for CRC cell exosomes and Exoquick for serum exosomes) do not allow the discrimination of exosomes from high-density and low-density lipoproteins (HDL and LDL, respectively), because all of these particles have a similar density.[70]

RNA Isolation from Cell Lines and Exosomes

Total RNA was extracted from cell lines and exosomes with TriZol (Invitrogen), according to the manufacturer's instructions, and quantified by GenQuant pro spectrophotometer (Biochrom) and Qubit fluorescence quantification system (Invitrogen).

PCR Primer Design

By literature mining, we selected for this study 17 lncRNAs involved in CRC or other tumors and regulating cancer-related processes, such as cell cycle, apoptosis, gene expression, and splicing. We designed specific PCR primers for the selected lncRNAs and two housekeeping genes used for normalization: PPIA (peptidylprolyl isomerase A) and GAPDH (glyceraldehyde 3-phosphate dehydrogenase).[71] Primer

design was performed by using the online tool Primer-Blast (https://www.ncbi.nlm.nih.gov/tools/primer-blast/). Primer pairs are shown in Table S9. PCR primers for 31 circRNAs were retrieved from the paper published by Memczak and colleagues.[4]

NcRNA Expression Analysis by Real-Time PCR

We investigated the expression of selected lncRNAs and circRNAs in CRC patient tissues and serum exosomes and in CRC cell lines and exosomes through Real-Time PCR by using Power SYBR Green RNA-to-CT 1-Step kit (Applied Biosystems, Thermo Fisher Scientific, Waltham, MA), according to the manufacturer's instructions. All Real-Time PCR reactions were performed on a 7900HT Fast Real-Time PCR System (Applied Biosystems). DE lncRNAs and circRNAs were identified through SDS RQ Manager 1.2 software (Applied Biosystems); normalization was performed using PPIA and GAPDH for tumor tissues and serum exosomes, respectively. miRNA expression was assessed through TaqMan microRNA assays and TaqMan Universal Master Mix II, no UNG (Applied Biosystems);[72] miR-24 was used as endogenous control. Expression fold changes of DE ncRNAs were calculated by applying the $2^{-\Delta\Delta Ct}$ method. Statistical analysis was performed by paired two-tailed t test to compare tissue sample ΔCts, while unpaired two-tailed t test was used for ΔCts from the following: (1) serum exosomes, (2) U0126 treatment, and (3) UCA1 silencing. Statistical significance was established at a p-value $\leq$ 0.05.

ROC Curve Analysis

To evaluate the diagnostic accuracy of ncRNAs deregulated in patient serum exosomes, ROC curves were computed through the software MedCalc v15.11.4. The AUC and 95% CIs were calculated to assess the accuracy of sensitivity and specificity parameters and find the appropriate cutoff point. Statistical significance was established at a p-value $\leq$ 0.05. Combined ROC curves were also computed by using ΔCts calculated as $Ct_{ncRNA1} - Ct_{ncRNA2}$, combining two ncRNAs showing an opposite trend of expression.

CRC Cell Line Treatment with MAPK Inhibitor U0126

MAPK inhibition was achieved by treating the HCT-116 cell line with U0126, a highly selective ATP-non-competitive inhibitor of both Mek1 and Mek2 kinases. Cells (3.2×10^4 per well) were seeded in 24-well plates and cultured in serum starvation conditions (no FBS) for 24 hr; successively, cells were treated with 25 μM U0126 (MEK1/2 inhibitor, Merck, Darmstadt, Germany). Cells were exposed to drug for 6, 12, 24, and 48 hr. Control samples were treated with an equivalent volume of DMSO (solvent of the drug used for treatment). All experiments were performed in biological triplicates. Cell viability was assessed through the MTT [3-(4,5-dimethylthiazol-2-yl)-2,5-diphenyltetrazolium bromide] assay at 24 hr post-treatment.

Protein Extraction and Western Blot Analysis

MAPK pathway inhibition was verified by analyzing p-ERK levels through western analysis. We performed protein extraction through radioimmunoprecipitation assay (RIPA) buffer and quantification through Qubit. Blotting was performed by iBlot Dry Blotting System (Invitrogen). Proteins were detected using the ECL Plus Western Blotting detection (Amersham Biosciences, Little Chalfont, Buckinghamshire, UK). Membranes were incubated overnight with monoclonal antibodies to p44/42 MAPK (Erk1/2) and phospho-p44/p42 MAPK (Erk1/2) (Cell Signaling, Leiden, the Netherlands); beta-actin (Abcam, Cambridge, MA) was used as loading control. Bound antibodies were detected with peroxidase-labeled goat antirabbit IgG-horseradish peroxidase (HRP) secondary antibody (Santa Cruz Biotechnology, Dallas, TX).[73]

In Vitro Silencing of UCA1

In vitro silencing of lncRNA UCA1 was performed in the HCT-116 cell line through antisense oligonucleotide (ASO) technology. Cells (5×10^4 per well) were seeded in 24-well plates and simultaneously reverse transfected with 100 nM UCA1 specific Antisense LNA GapmeR (Exiqon, Vedbaek, Denmark) and Lipofectamine RNAiMAX Reagent (Invitrogen), according to the manufacturers' instructions. Cells treated with a scramble molecule were used as controls. All experiments were performed in biological triplicates. Cells were harvested at 24 and 48 hr after transfection and lysed with Trizol. Specific PCR primers were designed and used to evaluate the expression of UCA1 target mRNAs by Real-Time PCR (Table S9).

Computational Analysis

The results obtained from CRC biopsies were validated in independent cohorts represented by datasets of microarray experiments from GEO DataSets (https://www.ncbi.nlm.nih.gov/gds/). We reanalyzed the expression of DE lncRNAs in 14 CRC datasets; also, results obtained in serum exosomes were confirmed by analyzing a dataset on peripheral blood samples from CRC patients. All analyzed datasets are listed in Table S10. Potential TFBSs harbored on the UCA1 promoter and upstream regulatory region (1 kb) were retrieved from ENCODE tracks (i.e., DNase hypersensitive and chromatin immunoprecipitation sequencing [ChIP-seq] on TFs and H3K27Ac, H3K4me1, and H3K4me3 histone marks) mapped on the UCSC Genome Browser (https://genome.ucsc.edu/). The existence of a positive or negative correlation of expression between the identified TFs and UCA1 was evaluated in expression datasets showing increased expression of UCA1 in CRC tumor tissues compared to normal mucosa.

Prediction of miRNAs sponged by UCA1 was performed through miRcode (http://www.mircode.org/). MiRNA targets experimentally validated in CRC models were retrieved by StarBase (http://starbase.sysu.edu.cn/),[74] miRTarbase (http://mirtarbase.mbc.nctu.edu.tw/php/index.php),[75] and literature data mining; their expression in CRC was investigated in CRC datasets by Oncomine (https://www.oncomine.org/). MiRNAs showing binding sites within the UCA1 sequence and acting as tumor suppressors in CRC were selected for subsequent analysis. Among the experimentally validated targets of selected miRNAs, those characterized by a positive correlation of expression with UCA1 were selected (see also the Results). Correlation analysis (Pearson or Spearman correlation, p-value $\leq$ 0.05)

was performed on GEO and TCGA datasets. RNA-RNA interactions were predicted by using the IntaRNA tool (http://rna.informatik.uni-freiburg.de/IntaRNA/Input.jsp).[76] MiRNAs binding the 3′-UTRs of selected targets were predicted by microrna.org (http://34.236.212.39/microrna/home.do).[77] A molecular network was built and visualized by using the BisoGenet plug-in of Cytoscape 3.6 (http://www.cytoscape.org).[78,79] The FatiGO tool (http://v4.babelomics.org) was applied to determine the statistical over-representation (Fisher's exact test, p-value < 0.05) of Gene Ontology (GO), Kyoto Encyclopedia of Genes and Genomes (KEGG), Reactome, and BioCarta terms within the molecular network.

SUPPLEMENTAL INFORMATION

Supplemental Information includes three figures and ten tables and can be found with this article online at https://doi.org/10.1016/j.omtn.2018.05.009.

AUTHOR CONTRIBUTIONS

M.P., M.R., C.D.P., F.B., A. Cappellani, and A.B. designed and conceived the experiments. R.C. and A.M. obtained and characterized biological samples from patients. C.B. performed the experiments. C.B., D. Brex, A. Caponnetto, M.C., M.S., and D. Barbagallo contributed to the acquisition, analysis, and interpretation of data. M.P., C.B., and M.R. wrote the paper. All authors read and approved the manuscript.

CONFLICTS OF INTEREST

The authors declare no conflict of interest.

ACKNOWLEDGMENTS

This study was supported by the the University of Catania through Finanziamento della Ricerca 2014 and 2016/2018 Department Research Plan of University of Catania (second line of intervention). We wish to thank the Scientific Bureau of the University of Catania for language support.

REFERENCES

1. Ragusa, M., Barbagallo, C., Statello, L., Condorelli, A.G., Battaglia, R., Tamburello, L., Barbagallo, D., Di Pietro, C., and Purrello, M. (2015). Non-coding landscapes of colorectal cancer. World J. Gastroenterol. *21*, 11709–11739.

2. Gomes, A.Q., Nolasco, S., and Soares, H. (2013). Non-coding RNAs: multi-tasking molecules in the cell. Int. J. Mol. Sci. *14*, 16010–16039.

3. Chen, I., Chen, C.Y., and Chuang, T.J. (2015). Biogenesis, identification, and function of exonic circular RNAs. Wiley Interdiscip. Rev. RNA *6*, 563–579.

4. Memczak, S., Jens, M., Elefsinioti, A., Torti, F., Krueger, J., Rybak, A., Maier, L., Mackowiak, S.D., Gregersen, L.H., Munschauer, M., et al. (2013). Circular RNAs are a large class of animal RNAs with regulatory potency. Nature *495*, 333–338.

5. Barbagallo, D., Condorelli, A., Ragusa, M., Salito, L., Sammito, M., Banelli, B., Caltabiano, R., Barbagallo, G., Zappalà, A., Battaglia, R., et al. (2016). Dysregulated miR-671-5p / CDR1-AS / CDR1 / VSNL1 axis is involved in glioblastoma multiforme. Oncotarget *7*, 4746–4759.

6. Barbagallo, D., Caponnetto, A., Cirnigliaro, M., Brex, D., Barbagallo, C., D'Angeli, F., Morrone, A., Caltabiano, R., Barbagallo, G.M., Ragusa, M., et al. (2018). CircSMARCA5 Inhibits Migration of Glioblastoma Multiforme Cells by Regulating a Molecular Axis Involving Splicing Factors SRSF1/SRSF3/PTB. Int. J. Mol. Sci. *19*, E480.

7. Hansen, T.B., Jensen, T.I., Clausen, B.H., Bramsen, J.B., Finsen, B., Damgaard, C.K., and Kjems, J. (2013). Natural RNA circles function as efficient microRNA sponges. Nature *495*, 384–388.

8. Ragusa, M., Barbagallo, C., Brex, D., Caponnetto, A., Cirnigliaro, M., Battaglia, R., Barbagallo, D., Di Pietro, C., and Purrello, M. (2017). Molecular Crosstalking among Noncoding RNAs: A New Network Layer of Genome Regulation in Cancer. Int. J. Genomics *2017*, 4723193.

9. Kogure, T., Yan, I.K., Lin, W.L., and Patel, T. (2013). Extracellular Vesicle-Mediated Transfer of a Novel Long Noncoding RNA TUC339: A Mechanism of Intercellular Signaling in Human Hepatocellular Cancer. Genes Cancer *4*, 261–272.

10. Takahashi, K., Yan, I.K., Kogure, T., Haga, H., and Patel, T. (2014). Extracellular vesicle-mediated transfer of long non-coding RNA ROR modulates chemosensitivity in human hepatocellular cancer. FEBS Open Bio *4*, 458–467.

11. Gezer, U., Özgür, E., Cetinkaya, M., Isin, M., and Dalay, N. (2014). Long non-coding RNAs with low expression levels in cells are enriched in secreted exosomes. Cell Biol. Int. *38*, 1076–1079.

12. Lasda, E., and Parker, R. (2016). Circular RNAs Co-Precipitate with Extracellular Vesicles: A Possible Mechanism for circRNA Clearance. PLoS ONE *11*, e0148407.

13. Urbanelli, L., Magini, A., Buratta, S., Brozzi, A., Sagini, K., Polchi, A., Tancini, B., and Emiliani, C. (2013). Signaling pathways in exosomes biogenesis, secretion and fate. Genes (Basel) *4*, 152–170.

14. Théry, C., Boussac, M., Véron, P., Ricciardi-Castagnoli, P., Raposo, G., Garin, J., and Amigorena, S. (2001). Proteomic analysis of dendritic cell-derived exosomes: a secreted subcellular compartment distinct from apoptotic vesicles. J. Immunol. *166*, 7309–7318.

15. Kalluri, R., and LeBleu, V.S. (2016). Discovery of Double-Stranded Genomic DNA in Circulating Exosomes. Cold Spring Harb. Symp. Quant. Biol. *81*, 275–280.

16. Keller, S., Ridinger, J., Rupp, A.K., Janssen, J.W., and Altevogt, P. (2011). Body fluid derived exosomes as a novel template for clinical diagnostics. J. Transl. Med. *9*, 86.

17. Ludwig, A.K., and Giebel, B. (2012). Exosomes: small vesicles participating in intercellular communication. Int. J. Biochem. Cell Biol. *44*, 11–15.

18. Qi, P., Zhou, X.Y., and Du, X. (2016). Circulating long non-coding RNAs in cancer: current status and future perspectives. Mol. Cancer *15*, 39.

19. Siegel, R.L., Miller, K.D., and Jemal, A. (2016). Cancer statistics, 2016. CA Cancer J. Clin. *66*, 7–30.

20. Weng, M., Wu, D., Yang, C., Peng, H., Wang, G., Wang, T., and Li, X. (2017). Noncoding RNAs in the development, diagnosis, and prognosis of colorectal cancer. Transl. Res. *181*, 108–120.

21. Yang, L., Xu, L., Wang, Q., Wang, M., and An, G. (2016). Dysregulation of long non-coding RNA profiles in human colorectal cancer and its association with overall survival. Oncol. Lett. *12*, 4068–4074.

22. Wang, J., Song, Y.X., Ma, B., Wang, J.J., Sun, J.X., Chen, X.W., Zhao, J.H., Yang, Y.C., and Wang, Z.N. (2015). Regulatory Roles of Non-Coding RNAs in Colorectal Cancer. Int. J. Mol. Sci. *16*, 19886–19919.

23. Mhaidat, N.M., Alali, F.Q., Matalqah, S.M., Matalka, I.I., Jaradat, S.A., Al-Sawalha, N.A., and Thorne, R.F. (2009). Inhibition of MEK sensitizes paclitaxel-induced apoptosis of human colorectal cancer cells by downregulation of GRP78. Anticancer Drugs *20*, 601–606.

24. Xiang, J.F., Yin, Q.F., Chen, T., Zhang, Y., Zhang, X.O., Wu, Z., Zhang, S., Wang, H.B., Ge, J., Lu, X., et al. (2014). Human colorectal cancer-specific CCAT1-L lncRNA regulates long-range chromatin interactions at the MYC locus. Cell Res. *24*, 513–531.

25. Ling, H., Spizzo, R., Atlasi, Y., Nicoloso, M., Shimizu, M., Redis, R.S., Nishida, N., Gafà, R., Song, J., Guo, Z., et al. (2013). CCAT2, a novel noncoding RNA mapping to 8q24, underlies metastatic progression and chromosomal instability in colon cancer. Genome Res. *23*, 1446–1461.

26. Kogo, R., Shimamura, T., Mimori, K., Kawahara, K., Imoto, S., Sudo, T., Tanaka, F., Shibata, K., Suzuki, A., Komune, S., et al. (2011). Long noncoding RNA HOTAIR regulates polycomb-dependent chromatin modification and is associated with poor prognosis in colorectal cancers. Cancer Res. *71*, 6320–6326.

27. Wu, Z.H., Wang, X.L., Tang, H.M., Jiang, T., Chen, J., Lu, S., Qiu, G.Q., Peng, Z.H., and Yan, D.W. (2014). Long non-coding RNA HOTAIR is a powerful predictor of

metastasis and poor prognosis and is associated with epithelial-mesenchymal transition in colon cancer. Oncol. Rep. *32*, 395–402.

28. Ren, Z., Wang, W., and Li, J. (2016). Identifying molecular subtypes in human colon cancer using gene expression and DNA methylation microarray data. Int. J. Oncol. *48*, 690–702.

29. Tao, K., Yang, J., Hu, Y., Sun, Y., Tan, Z., Duan, J., Zhang, F., Yan, H., and Deng, A. (2015). Clinical significance of urothelial carcinoma associated 1 in colon cancer. Int. J. Clin. Exp. Med. *8*, 21854–21860.

30. Ni, B., Yu, X., Guo, X., Fan, X., Yang, Z., Wu, P., Yuan, Z., Deng, Y., Wang, J., Chen, D., and Wang, L. (2015). Increased urothelial cancer associated 1 is associated with tumor proliferation and metastasis and predicts poor prognosis in colorectal cancer. Int. J. Oncol. *47*, 1329–1338.

31. Lin, P.C., Huang, H.D., Chang, C.C., Chang, Y.S., Yen, J.C., Lee, C.C., Chang, W.H., Liu, T.C., and Chang, J.G. (2016). Long noncoding RNA TUG1 is downregulated in non-small cell lung cancer and can regulate CELF1 on binding to PRC2. BMC Cancer *16*, 583.

32. Fan, S., Yang, Z., Ke, Z., Huang, K., Liu, N., Fang, X., and Wang, K. (2017). Downregulation of the long non-coding RNA TUG1 is associated with cell proliferation, migration, and invasion in breast cancer. Biomed. Pharmacother. *95*, 1636–1643.

33. Li, J., Zhang, M., An, G., and Ma, Q. (2016). LncRNA TUG1 acts as a tumor suppressor in human glioma by promoting cell apoptosis. Exp. Biol. Med. (Maywood) *241*, 644–649.

34. Han, Y., Wu, Z., Wu, T., Huang, Y., Cheng, Z., Li, X., Sun, T., Xie, X., Zhou, Y., and Du, Z. (2016). Tumor-suppressive function of long noncoding RNA MALAT1 in glioma cells by downregulation of MMP2 and inactivation of ERK/MAPK signaling. Cell Death Dis. *7*, e2123.

35. Xu, S., Sui, S., Zhang, J., Bai, N., Shi, Q., Zhang, G., Gao, S., You, Z., Zhan, C., Liu, F., and Pang, D. (2015). Downregulation of long noncoding RNA MALAT1 induces epithelial-to-mesenchymal transition via the PI3K-AKT pathway in breast cancer. Int. J. Clin. Exp. Pathol. *8*, 4881–4891.

36. Ji, Q., Zhang, L., Liu, X., Zhou, L., Wang, W., Han, Z., Sui, H., Tang, Y., Wang, Y., Liu, N., et al. (2014). Long non-coding RNA MALAT1 promotes tumour growth and metastasis in colorectal cancer through binding to SFPQ and releasing oncogene PTBP2 from SFPQ/PTBP2 complex. Br. J. Cancer *111*, 736–748.

37. Zheng, H.T., Shi, D.B., Wang, Y.W., Li, X.X., Xu, Y., Tripathi, P., Gu, W.L., Cai, G.X., and Cai, S.J. (2014). High expression of lncRNA MALAT1 suggests a biomarker of poor prognosis in colorectal cancer. Int. J. Clin. Exp. Pathol. *7*, 3174–3181.

38. Sun, J., Ding, C., Yang, Z., Liu, T., Zhang, X., Zhao, C., and Wang, J. (2016). The long non-coding RNA TUG1 indicates a poor prognosis for colorectal cancer and promotes metastasis by affecting epithelial-mesenchymal transition. J. Transl. Med. *14*, 42.

39. Wang, L., Zhao, Z., Feng, W., Ye, Z., Dai, W., Zhang, C., Peng, J., and Wu, K. (2016). Long non-coding RNA TUG1 promotes colorectal cancer metastasis via EMT pathway. Oncotarget *7*, 51713–51719.

40. Feng, T., Shao, F., Wu, Q., Zhang, X., Xu, D., Qian, K., Xie, Y., Wang, S., Xu, N., Wang, Y., and Qi, C. (2016). miR-124 downregulation leads to breast cancer progression via LncRNA-MALAT1 regulation and CDK4/E2F1 signal activation. Oncotarget *7*, 16205–16216.

41. Li, J., An, G., Zhang, M., and Ma, Q. (2016). Long non-coding RNA TUG1 acts as a miR-26a sponge in human glioma cells. Biochem. Biophys. Res. Commun. *477*, 743–748.

42. Nissan, A., Stojadinovic, A., Mitrani-Rosenbaum, S., Halle, D., Grinbaum, R., Roistacher, M., Bochem, A., Dayanc, B.E., Ritter, G., Gomceli, I., et al. (2012). Colon cancer associated transcript-1: a novel RNA expressed in malignant and pre-malignant human tissues. Int. J. Cancer *130*, 1598–1606.

43. Alaiyan, B., Ilyayev, N., Stojadinovic, A., Izadjoo, M., Roistacher, M., Pavlov, V., Tzivin, V., Halle, D., Pan, H., Trink, B., et al. (2013). Differential expression of colon cancer associated transcript1 (CCAT1) along the colonic adenoma-carcinoma sequence. BMC Cancer *13*, 196.

44. He, X., Tan, X., Wang, X., Jin, H., Liu, L., Ma, L., Yu, H., and Fan, Z. (2014). C-Myc-activated long noncoding RNA CCAT1 promotes colon cancer cell proliferation and invasion. Tumour Biol. *35*, 12181–12188.

45. Tang, W., Ji, M., He, G., Yang, L., Niu, Z., Jian, M., Wei, Y., Ren, L., and Xu, J. (2017). Silencing CDR1as inhibits colorectal cancer progression through regulating microRNA-7. OncoTargets Ther. *10*, 2045–2056.

46. Knickelbein, K., and Zhang, L. (2015). Mutant KRAS as a critical determinant of the therapeutic response of colorectal cancer. Genes Dis. *2*, 4–12.

47. Zenonos, K., and Kyprianou, K. (2013). RAS signaling pathways, mutations and their role in colorectal cancer. World J. Gastrointest. Oncol. *5*, 97–101.

48. Piwien Pilipuk, G., Galigniana, M.D., and Schwartz, J. (2003). Subnuclear localization of C/EBP beta is regulated by growth hormone and dependent on MAPK. J. Biol. Chem. *278*, 35668–35677.

49. Tang, Q.Q., Grønborg, M., Huang, H., Kim, J.W., Otto, T.C., Pandey, A., and Lane, M.D. (2005). Sequential phosphorylation of CCAAT enhancer-binding protein beta by MAPK and glycogen synthase kinase 3beta is required for adipogenesis. Proc. Natl. Acad. Sci. USA *102*, 9766–9771.

50. Piwien-Pilipuk, G., MacDougald, O., and Schwartz, J. (2002). Dual regulation of phosphorylation and dephosphorylation of C/EBPbeta modulate its transcriptional activation and DNA binding in response to growth hormone. J. Biol. Chem. *277*, 44557–44565.

51. Rask, K., Thörn, M., Pontén, F., Kraaz, W., Sundfeldt, K., Hedin, L., and Enerbäck, S. (2000). Increased expression of the transcription factors CCAAT-enhancer binding protein-beta (C/EBBeta) and C/EBzeta (CHOP) correlate with invasiveness of human colorectal cancer. Int. J. Cancer *86*, 337–343.

52. Sun, D., Wang, C., Long, S., Ma, Y., Guo, Y., Huang, Z., Chen, X., Zhang, C., Chen, J., and Zhang, J. (2015). C/EBP-β-activated microRNA-223 promotes tumour growth through targeting RASA1 in human colorectal cancer. Br. J. Cancer *112*, 1491–1500.

53. Zheng, Q., Bao, C., Guo, W., Li, S., Chen, J., Chen, B., Luo, Y., Lyu, D., Li, Y., Shi, G., et al. (2016). Circular RNA profiling reveals an abundant circHIPK3 that regulates cell growth by sponging multiple miRNAs. Nat. Commun. *7*, 11215.

54. Li, Y., Zheng, Q., Bao, C., Li, S., Guo, W., Zhao, J., Chen, D., Gu, J., He, X., and Huang, S. (2015). Circular RNA is enriched and stable in exosomes: a promising biomarker for cancer diagnosis. Cell Res. *25*, 981–984.

55. Isin, M., Ozgur, E., Cetin, G., Erten, N., Aktan, M., Gezer, U., and Dalay, N. (2014). Investigation of circulating lncRNAs in B-cell neoplasms. Clin. Chim. Acta *431*, 255–259.

56. Ma, B., Li, M., Zhang, L., Huang, M., Lei, J.B., Fu, G.H., Liu, C.X., Lai, Q.W., Chen, Q.Q., and Wang, Y.L. (2016). Upregulation of long non-coding RNA TUG1 correlates with poor prognosis and disease status in osteosarcoma. Tumour Biol. *37*, 4445–4455.

57. Galamb, O., Sipos, F., Solymosi, N., Spisák, S., Krenács, T., Tóth, K., Tulassay, Z., and Molnár, B. (2008). Diagnostic mRNA expression patterns of inflamed, benign, and malignant colorectal biopsy specimen and their correlation with peripheral blood results. Cancer Epidemiol. Biomarkers Prev. *17*, 2835–2845.

58. Ragusa, M., Barbagallo, C., Cirnigliaro, M., Battaglia, R., Brex, D., Caponnetto, A., Barbagallo, D., Di Pietro, C., and Purrello, M. (2017). Asymmetric RNA Distribution among Cells and Their Secreted Exosomes: Biomedical Meaning and Considerations on Diagnostic Applications. Front. Mol. Biosci. *4*, 66.

59. Borrebaeck, C.A. (2017). Precision diagnostics: moving towards protein biomarker signatures of clinical utility in cancer. Nat. Rev. Cancer *17*, 199–204.

60. Sgroi, D.C. (2009). The HOXB13:IL17BR gene-expression ratio: a biomarker providing information above and beyond tumor grade. Biomarkers Med. *3*, 99–102.

61. Ma, X.J., Wang, Z., Ryan, P.D., Isakoff, S.J., Barmettler, A., Fuller, A., Muir, B., Mohapatra, G., Salunga, R., Tuggle, J.T., et al. (2004). A two-gene expression ratio predicts clinical outcome in breast cancer patients treated with tamoxifen. Cancer Cell *5*, 607–616.

62. Nie, W., Ge, H.J., Yang, X.Q., Sun, X., Huang, H., Tao, X., Chen, W.S., and Li, B. (2016). LncRNA-UCA1 exerts oncogenic functions in non-small cell lung cancer by targeting miR-193a-3p. Cancer Lett. *371*, 99–106.

63. Tuo, Y.L., Li, X.M., and Luo, J. (2015). Long noncoding RNA UCA1 modulates breast cancer cell growth and apoptosis through decreasing tumor suppressive miR-143. Eur. Rev. Med. Pharmacol. Sci. *19*, 3403–3411.

64. Zheng, J., Yi, D., Liu, Y., Wang, M., Zhu, Y., and Shi, H. (2017). Long nonding RNA UCA1 regulates neural stem cell differentiation by controlling miR-1/Hes1 expression. Am. J. Transl. Res. *9*, 3696–3704.

65. Luo, J., Chen, J., Li, H., Yang, Y., Yun, H., Yang, S., and Mao, X. (2017). LncRNA UCA1 promotes the invasion and EMT of bladder cancer cells by regulating the miR-143/HMGB1 pathway. Oncol. Lett. *14*, 5556–5562.

66. Wu, H., and Zhou, C. (2018). Long non-coding RNA UCA1 promotes lung cancer cell proliferation and migration via microRNA-193a/HMGB1 axis. Biochem Biophys Res Commun. *496*, 738–745.

67. Sun, Y., Jin, J.G., Mi, W.Y., Zhang, S.R., Meng, Q., and Zhang, S.T. (2018). Long Noncoding RNA UCA1 Targets miR-122 to Promote Proliferation, Migration, and Invasion of Glioma Cells. Oncol Res. *26*, 103–110.

68. Zuo, Z.K., Gong, Y., Chen, X.H., Ye, F., Yin, Z.M., Gong, Q.N., and Huang, J.S. (2017). TGFβ1-Induced LncRNA UCA1 Upregulation Promotes Gastric Cancer Invasion and Migration. DNA Cell Biol. *36*, 159–167.

69. Ragusa, M., Statello, L., Maugeri, M., Barbagallo, C., Passanisi, R., Alhamdani, M.S., Li Destri, G., Cappellani, A., Barbagallo, D., Scalia, M., et al. (2014). Highly skewed distribution of miRNAs and proteins between colorectal cancer cells and their exosomes following Cetuximab treatment: biomolecular, genetic and translational implications. Oncoscience *1*, 132–157.

70. Popović, M., and de Marco, A. (2018). Canonical and selective approaches in exosome purification and their implications for diagnostic accuracy. Transl. Cancer Res. *7*, S209–S225.

71. Vandesompele, J., De Preter, K., Pattyn, F., Poppe, B., Van Roy, N., De Paepe, A., and Speleman, F. (2002). Accurate normalization of real-time quantitative RT-PCR data by geometric averaging of multiple internal control genes. Genome Biol. *3*, RESEARCH0034.

72. Maugeri, M., Barbagallo, D., Barbagallo, C., Banelli, B., Di Mauro, S., Purrello, F., Magro, G., Ragusa, M., Di Pietro, C., Romani, M., and Purrello, M. (2016). Altered expression of miRNAs and methylation of their promoters are correlated in neuroblastoma. Oncotarget *7*, 83330–83341.

73. Ragusa, M., Statello, L., Maugeri, M., Majorana, A., Barbagallo, D., Salito, L., Sammito, M., Santonocito, M., Angelica, R., Cavallaro, A., et al. (2012). Specific alterations of the microRNA transcriptome and global network structure in colorectal cancer after treatment with MAPK/ERK inhibitors. J. Mol. Med. (Berl.) *90*, 1421–1438.

74. Li, J.H., Liu, S., Zhou, H., Qu, L.H., and Yang, J.H. (2014). starBase v2.0: decoding miRNA-ceRNA, miRNA-ncRNA and protein-RNA interaction networks from large-scale CLIP-Seq data. Nucleic Acids Res. *42*, D92–D97.

75. Chou, C.H., Shrestha, S., Yang, C.D., Chang, N.W., Lin, Y.L., Liao, K.W., Huang, W.C., Sun, T.H., Tu, S.J., Lee, W.H., et al. (2018). miRTarBase update 2018: a resource for experimentally validated microRNA-target interactions. Nucleic Acids Res. *46* (D1), D296–D302.

76. Mann, M., Wright, P.R., and Backofen, R. (2017). IntaRNA 2.0: enhanced and customizable prediction of RNA-RNA interactions. Nucleic Acids Res. *45* (W1), W435–W439.

77. Betel, D., Wilson, M., Gabow, A., Marks, D.S., and Sander, C. (2008). The microRNA.org resource: targets and expression. Nucleic Acids Res. *36*, D149–D153.

78. Ragusa, M., Avola, G., Angelica, R., Barbagallo, D., Guglielmino, M.R., Duro, L.R., Majorana, A., Statello, L., Salito, L., Consoli, C., et al. (2010). Expression profile and specific network features of the apoptotic machinery explain relapse of acute myeloid leukemia after chemotherapy. BMC Cancer *10*, 377.

79. Di Pietro, C., Ragusa, M., Barbagallo, D., Duro, L.R., Guglielmino, M.R., Majorana, A., Angelica, R., Scalia, M., Statello, L., Salito, L., et al. (2009). The apoptotic machinery as a biological complex system: analysis of its omics and evolution, identification of candidate genes for fourteen major types of cancer, and experimental validation in CML and neuroblastoma. BMC Med. Genomics *2*, 20.

OMTN, Volume *12*

Supplemental Information

LncRNA UCA1, Upregulated in CRC Biopsies and Downregulated in Serum Exosomes, Controls mRNA Expression by RNA-RNA Interactions

Cristina Barbagallo, Duilia Brex, Angela Caponnetto, Matilde Cirnigliaro, Marina Scalia, Antonio Magnano, Rosario Caltabiano, Davide Barbagallo, Antonio Biondi, Alessandro Cappellani, Francesco Basile, Cinzia Di Pietro, Michele Purrello, and Marco Ragusa

Supplemental Figure 1: Expression of lncRNAs and circRNAs in CRC cell lines HCT-116 and Caco-2.

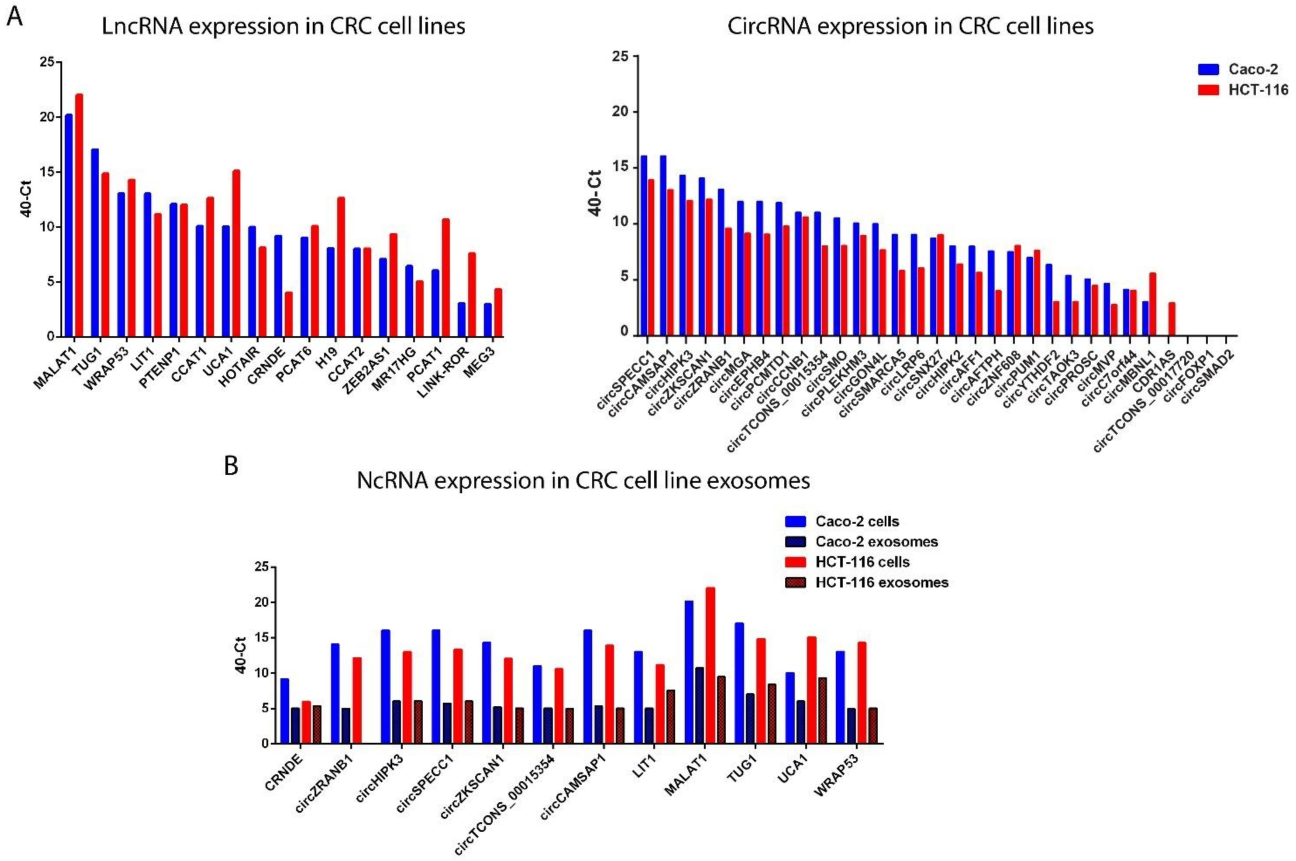

The most abundant ncRNAs in CRC cells were investigated in their exosomes. Expression is shown as 40-Ct.

Supplemental Figure 2: Effects of MAPK inhibition.

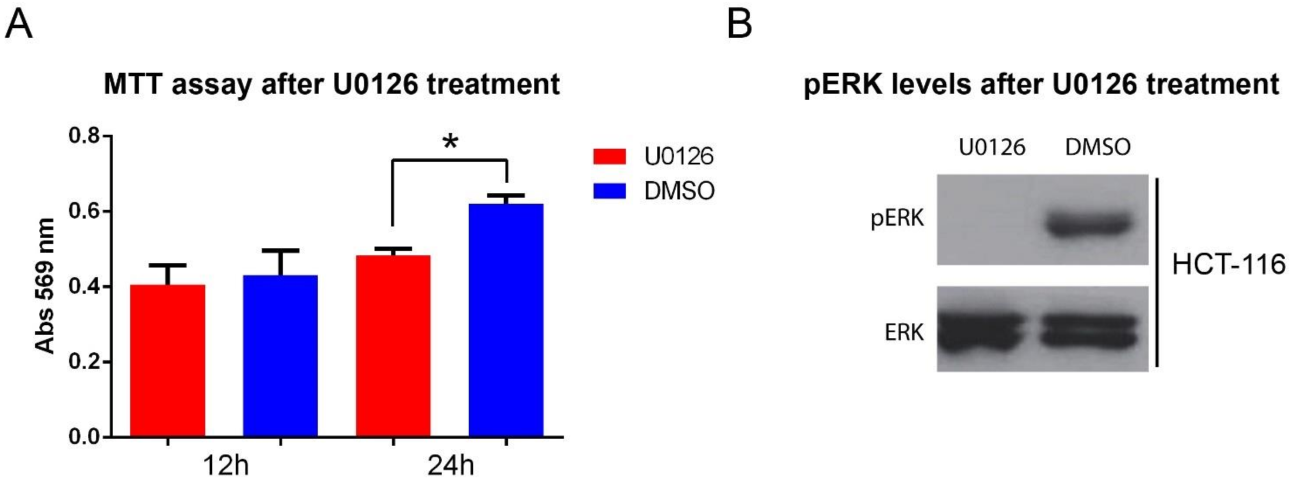

A) treatment with U0126 significantly reduced cell viability, measured by MTT assay 24 hours after treatment; cells treated with DMSO were used as controls; error bars represent standard deviation. *: p-value = 4.96E-7. B) Western Blot showing Erk and p-Erk levels in HCT-116 cells treated with MAPK inhibitor U0126 compared to control cells treated with DMSO at 12 hours post treatment: MAPK inhibition is showed by reduced levels of p-Erk compared to Erk.

Supplemental Figure 3: Biological processes controlled by the ceRNA network regulated by UCA1.

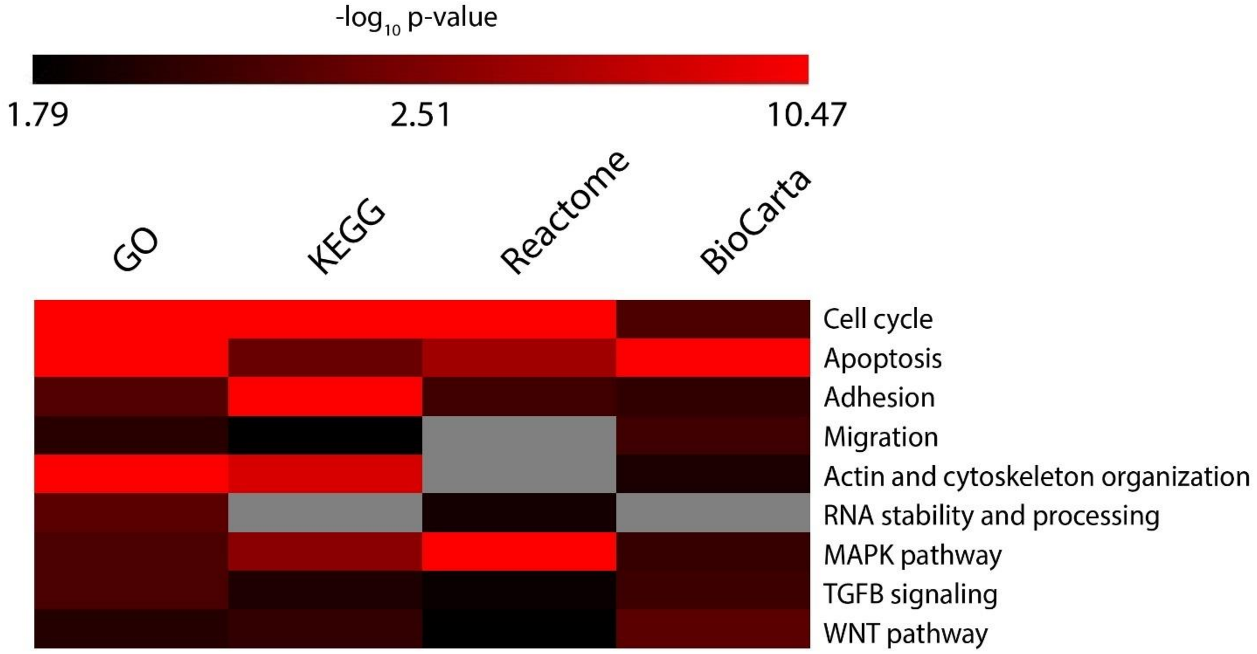

Over-represented biological functions of the molecular network arose from interactions among ANLN, BIRC5, IPO7, KIF23, and KIF2A, retrieved from different annotation databases (GO, KEGG, Reactome, BioCarta). Data are shown as -log10 of p-values for each biological process, as shown in the colour coded bar.

Supplemental Table 1: Analysis of CRC GEO datasets.

	**GEO dataset ID**	**Pearson (p-value)**
MALAT1	GSE37892	-0.475 (1.10E-08)
	GSE35896	-0.4 (1.30E-03)
	GSE8671	-0.33 (7.70E-03)
	GSE13294	-0.24 (2.70E-03)
	GSE4554	-0.23 (3.00E-02)
	GSE33114	-0.217 (2.00E-02)
	GSE14333	0.477 (6.80E-18)
	GSE23878	0.523 (2.20E-05)
	GSE39395	0.616 (1.30E-03)
	GSE3629	0.95 (2.50E-62)
UCA1	GSE33114	-0.272 (4.40E-03)
	GSE14333	-0.18 (2.10E-03)
	GSE2109	0.375 (2.00E-02)
	GSE8671	0.578 (5.80E-07)
	GSE3629	0.69 (9.90E-19)

Expression correlation between ncRNAs deregulated at 6 hours after U0126 treatment and HSPA5, a transcriptional target of p-Erk. The Pearson coefficient and its p-value (between brackets) are shown.

Supplemental Table 2: Hypothetical transcription factors regulating UCA1 expression.

	**CEBPB**	**TEAD4**	**TFAP2A**	**TFAP2C**
**TFBS Position**	chr19:15938912-15939187	chr19:15939692-15939850	chr19:15939115-15939748	chr19:15939109-15939583
**Size (bp)**	276	159	634	475
**Position from TSS (bp)**	-845	-65	-642	-648
**GSE8671**	FC **1.74 (<0.00001)**	**3.5 (<0.00001)**	**1.5 (0.01)**	**2.24 (0.0002)**
	**0.65 (<0.00001)**	**0.7 (<0.00001)**	**0.5 (0.00002)**	0.15 (0.22)
**GSE9348**	**1.19 (<0.00001)**	**1.53 (<0.00001)**	**1.31 (0.00009)**	1.08 (0.23)
	**0.26 (0.02)**	**0.25 (0.01)**	0.18 (0.1)	0.09 (0.4)
**GSE20842**	**1.15 (<0.00001)**	**1.2 (<0.00001)**	**1.07 (<0.00001)**	**1.01 (<0.00001)**
	**0.4 (<0.00001)**	**0.61 (<0.00001)**	**0.59 (<0.00001)**	**0.37 (<0.00001)**
**GSE20916**	**1.1 (<0.00001)**	**1.71 (<0.00001)**	**1.01 (0.01)**	**1.02 (0.002)**
	**0.32 (0.0006)**	**0.35 (0.0001)**	**0.25 (0.007)**	**0.22 (0.02)**
**TCGA**	**3.08 (<0.00001)**	**3.62 (<0.00001)**	**2.24 (<0.00001)**	**2.33 (<0.00001)**
	**0.23 (0.001)**	**0.24 (0.001)**	**0.26 (0.0004)**	**0.20 (0.005)**

For each TFB, the genomic position, the size of the bound region and its distance from the transcription start site (TSS) are shown. The expression of TFs was investigated in CRC datasets showing increased expression of UCA1 in tumor tissues compared with normal mucosa. For each gene, fold change (FC), Pearson coefficient (Pc), and their p-value (between brackets) are shown. Statistically significant results are highlighted in bold.

Supplemental Table 3: Expression of miRNAs hypothetically sponged by UCA1 in CRC tissues compared to normal mucosa from GEO datasets.

	**GSE108153**	**GSE38389**	**GSE41655**	**GSE73487**	**GSE30454**	**GSE18392**	**GSE10259**	**GSE53339**
**miR-135a-5p**	1 (0.48)	**-1.01 (0.01)**	-1.17 (0.16)	1.06 (0.17)	**-3.28 (4.15E-10)**	**-1.4 (0.04)**		-1.22 (0.19)
**miR-143-3p**	**-4.77 (0.006)**	**-1.78 (5.77E-06)**	**-2.6 (1-79E-11)**	**-1.61 (0.002)**	**-1.21 (2.11E-05)**	1 (0.21)	**-2.45 (0.0009)**	**-13.61 (0.007)**
**miR-214-3p**	**1.28 (0.05)**	1 (0.4)	**-1.81 (0.003)**	**-1.33 (0.008)**	**-1.19 (0.01)**	1 (0.46)	1 (0.43)	**-1.17 (0.05)**
**miR-1271-5p**	**-1.54 (0.006)**	1 (0.36)		-1.09 (0.1)	**-1.81 (0.001)**			

Fold change and p-value (between brackets) are shown. Statistically significant results are highlighted in bold.

Supplemental Table 4: miRNAs and mRNAs selected as hypothetical targets of UCA1.

**miRNA targets**	**miR-135a (2)**	**miR-143 (4)**	**miR-214 (2)**	**miR-1271 (1)**
**ANLN**	x		x	x
**BIRC5**	x	x		
**BZW2**	x			
**CD46**	x	x	x	
**DEK**	x		x	
**DNMT3A**		x		
**HMMR**		x		x
**IPO7**			x	
**KIF2A**				x
**KIF23**	x	x		x
**MACC1**		x		
**MMP7**		x		
**MYC**	x			
**TRIP13**	x			

The number of miRNA binding sites lying within UCA1 sequence is shown for each miRNA between brackets; mRNA targets of each miRNA are indicated with "x".

Supplemental Table 5: **Expression of TFs and miRNA targets in CRC datasets.**

	**GSE8671**		**GSE9348**		**GSE20842**		**GSE20916**		**TCGA**	
	**FC (p-val.)**	**Pc (p-val.)**	**FC (p-val.)**	**Pc (p-val.)**	**FC (p-val.)**	**Pc (p-val.)**	**FC (p-val.)**	**Pc (p-val.)**	**FC (p-val.)**	**Pc (p-val.)**
ANLN	**4.3 (<0.00001)**	**0.51 (0.00001)**	**1.3 (<0.00001)**	0.18 (0.1)	**1.23 (<0.00001)**	**0.63 (0.00001)**	**1.55 (<0.00001)**	**0.29 (0.002)**	**2.24 (<0.00001)**	**0.23 (0.002)**
BIRC5	**3.12 (<0.00001)**	**0.51 (0.00001)**	**1.24 (<0.00001)**	**0.23 (0.03)**	\	\	**1.41 (<0.00001)**	**0.28 (0.003)**	**1.85 (<0.00001)**	**0.19 (0.008)**
BZW2	**1.57 (<0.00001)**	**0.46 (0.0001)**	**1.07 (<0.00001)**	0.1 (0.33)	**1.05 (<0.00001)**	**0.56 (< 0.00001)**	**1.07 (<0.00001)**	**0.32 (0.0006)**	**2.2 (<0.00001)**	**0.37 (<0.00001)**
CD46	**1.38 (<0.00001)**	**0.39 (0.001)**	**1.03 (0.0001)**	0.15 (0.17)	1 (0.12)	0.10 (0.22)	**1.07 (<0.00001)**	**0.4 (0.00002)**	**3.2 (<0.00001)**	**0.19 (0.01)**
CEBPB	**1.74 (<0.00001)**	**0.65 (<0.00001)**	**1.19 (<0.00001)**	**0.26 (0.02)**	**1.15 (<0.00001)**	**0.4 (<0.00001)**	**1.1 (<0.00001)**	**0.32 (0.0006)**	**3.08 (<0.00001)**	**0.23 (0.001)**
DEK	**1.39 (<0.00001)**	**0.58 (<0.00001)**	**1.04 (0.0007)**	0.12 (0.2)	1.05 (<0.00001)	0.511 (<0.00001)	1.06 (<0.00001)	0.26 (0.007)	2.64 (<0.00001)	0.11 (0.14)
DNMT3A	**1.68 (<0.00001)**	**0.37 (0.002)**	**1.2 (<0.00001)**	0.13 (0.22)	**1.05 (<0.00001)**	**0.37 (<0.00001)**	**1.13 (<0.00001)**	**0.38 (0.008)**	**1.15 (0.05)**	**0.15 (0.04)**
HMMR	**2.26 (<0.00001)**	**0.41 (0.0006)**	**1.15 (<0.00001)**	0.08 (0.44)	**1.07 (<0.00001)**	**0.45 (<0.00001)**	**1.18 (<0.00001)**	**0.22 (0.02)**	**1.97 (<0.00001)**	**0.27 (0.0002)**
IPO7	**2.02 (<0.00001)**	**0.57 (<0.00001)**	**1.06 (<0.00001)**	0.34 (0.25)	**1.03 (<0.00001)**	**0.23 (0.006)**	**1.1 (<0.00001)**	**0.34 (0.0003)**	**1.31 (0.0005)**	0.08 (0.25)
KIF23	**3.49 (<0.00001)**	**0.51 (0.00001)**	**1.15 (<0.00001)**	0.04 (0.69)	**1.11 (<0.00001)**	**0.47 (<0.00001)**	**1.45 (<0.00001)**	0.17 (0.07)	**1.74 (<0.00001)**	0.12 (0.11)
KIF2A	**1.26 (<0.00001)**	**0.3 (0.01)**	1.01 (0.38)	0.1 (0.37)	**1.02 (0.004)**	**0.34 (0.00007)**	**1.05 (0.001)**	0.12 (0.19)	**1.38 (0.001)**	0.09 (0.19)
MACC1	**1.9 (<0.00001)**	**0.35 (0.004)**	**1.34 (<0.00001)**	**0.27 (0.001)**	**1.17 (<0.00001)**	**0.58 (<0.00001)**	**1.34 (<0.00001)**	**0.35 (0.0002)**	**3.4 (<0.00001)**	**0.32 (0.00001)**

MMP7	**40 (0.00001)**	**0.27 (0.02)**	**1.64 (<0.00001)**	**0.37 (0.0006)**	**1.74 (<0.00001)**	**0.69 (<0.00001)**	**3.39 (<0.00001)**	0.47 (0.47)	**6.47 (<0.00001)**	**0.43 (<0.00001)**
MYC	**3.13 (<0.00001)**	**0.6 (< 0.00001)**	**1.22 (<0.00001)**	**0.35 (0.001)**	**1.06 (<0.00001)**	**0.54 (<0.00001)**	**1.21 (<0.00001)**	**0.36 (0.0001)**	**2.27 (<0.00001)**	**0.4 (<0.00001)**
TEAD4	**3.5 (<0.00001)**	**0.7 (<0.00001)**	**1.53 (<0.00001)**	**0.25 (0.01)**	**1.2 (<0.00001)**	**0.61 (<0.00001)**	**1.71 (<0.00001)**	**0.35 (0.0001)**	**3.62 (<0.00001)**	**0.24 (0.001)**
TFAP2A	**1.5 (0.01)**	**0.5 (0.00002)**	**1.31 (0.00009)**	0.18 (0.1)	**1.07 (<0.00001)**	**0.59 (<0.00001)**	**1.01 (0.01)**	**0.25 (0.007)**	**2.24 (<0.00001)**	**0.26 (0.0004)**
TFAP2C	**2.24 (0.0002)**	0.15 (0.22)	1.08 (0.23)	0.09 (0.4)	**1.01 (<0.00001)**	**0.37 (<0.00001)**	**1.02 (0.002)**	**0.22 (0.02)**	**2.33 (<0.00001)**	**0.20 (0.005)**
TRIP13	**3.59 (<0.00001)**	**0.52 (< 0.00001)**	**1.25 (<0.00001)**	**0.21 (0.05)**	**1.17 (<0.00001)**	**0.6 (<0.00001)**	**1.47 (<0.00001)**	**0.35 (0.0002)**	**3.56 (<0.00001)**	**0.33 (<0.00001)**
UCA1	**4.3 (<0.00001)**	\	**1.13 (0.002)**	\	**1.27 (<0.00001)**	\	**1.74 (<0.00001)**	\	**2.09 (<0.00001)**	\

TFs and miRNA targets of UCA1 showed increased expression in tumor tissues compared to normal mucosa. For each gene, fold change (FC), Pearson coefficient (Pc), and their p-value (between brackets) are shown. Statistically significant results are highlighted in bold.

Supplemental Table 6: **MiRNAs binding the 3'-UTRs of selected miRNA targets were predicted by microrna.org.**

	**ANLN**	**BIRC5**	**IPO7**	**KIF2A**	**KIF23**	**Expression in CRC**
**hsa-let-7a**				388		down
**hsa-let-7d**				388		down
**hsa-let-7g**				388		down
**hsa-miR-1**				656		down
**miR-1271**					325	down
**hsa-miR-128**			267			down
**hsa-miR-135a**	569	648			40	down
**hsa-miR-135b**		647			40	up
**hsa-miR-15a**	794		803			down
**hsa-miR-15b**	794		805	646		down
**hsa-miR-16**	794		804			down
**hsa-miR-182**	820					up
**hsa-miR-183**				601	197	up
**hsa-miR-186**			450			down
**hsa-miR-190**				607		down
**hsa-miR-192**			473			down
**hsa-miR-195**	796		805			down
**hsa-miR-206**				658		down
**hsa-miR-214**			3			down
**hsa-miR-215**			473			down
**hsa-miR-217**	642					up
**hsa-miR-218**	615	620, 642				down
**hsa-miR-22**			303			down
**hsa-miR-224**			732, 761			up
**hsa-miR-300**					180	up
**hsa-miR-31**	821					up
**hsa-miR-326**					130	down
**hsa-miR-330-5p**					128	down

**hsa-miR-340**			455			down
**hsa-miR-342-3p**	568					down
**hsa-miR-361-5p**			479			down
**hsa-miR-376c**	800					down
**hsa-miR-377**	568	748				down
**hsa-miR-381**					180	down
**hsa-miR-410**				667		down
**hsa-miR-424**	795		804			up
**hsa-miR-448**	644					down
**hsa-miR-495**	853					down
**hsa-miR-497**	795		807			down
**hsa-miR-503**	793		804			up
**hsa-miR-539**					32	down
**hsa-miR-613**				660		down
**hsa-miR-653**					168	down
**hsa-miR-873**		1100				down

For each miRNA, the position of the binding site(s) on 3'-UTR and its expression in CRC datasets are shown.

Supplemental Table 7: expression of miRNAs binding the 3'-UTR in CRC expression datasets.

Supplemental Table 8: Functional enrichment analysis of the ceRNA network regulated by UCA1.

Supplemental Table 9: PCR primers.

Gene	Forward primer	Reverse primer
**ANLN**	TGTCTTCGTGGCCGATTTGA	TTTGAGGGGAGTGCTCTGAC
**BIRC5**	GAGGCTGGCTTCATCCACTG	CCTTTGCATGGGGTCGTCAT
**BZW2**	TTGGAGAAGGCATTTGAAGATGAAA	TCGCCAACTTTGTCTGCTCT
**CCAT1**	GGAAAGGTGCCGAGACATGA	GCCATACAGAGCCAACCTGG
**CCAT2**	CCTGCAGAGGGCACTAGACT	CCAGGGTCAGGCAATTGGTC
**CD46**	GGTGTTGCTGCTGTACTCCT	ACCAATGAGCTCCATAGCTTCAA
**CRNDE**	TCGATCGCGCTATTGTCATGG	CGCCTCGCTTAGACATTGGC
**DEK**	CAGCACCACCAAGAAGAATCAA	ACTCTTCATCTGTAGGGGGTTTC
**DNMT3A**	CGACCCCACCGGCCATA	CTCTGGTGAACGGTGCCTCT
**GAPDH**	GGCTAGCTGGCCCGATTT	AGGCGCCCAATACGACCAAA
**H19**	GAGTCGGCACACTATGGCTG	GTCCGGATTCAAAGGCCCAG
**HMMR**	ATGGTGCAAAGCCTTGAAGATG	GGCCGCTTTTTCCTGTAATGA
**HOTAIR**	CCCGGAGGTGCTCTCAATCA	ACTCCCCTACTGCAGGCTTC
**IPO7**	CAGGAGAAGATGCAGAGTGTCA	TCTAAGGCTGCTTCCACGAAT
**KIF2A**	GGGAGTTCCCCTCAGAGAGA	TGTGAAACAGCTTCGTGGAAAG
**KIF23**	TGGATATCAGCATCACGCACA	CTTCTGGCATGGCTTTCGAG
**LINC01764**	CTCCGGACTGCTTCAAGTGT	GTTATTCACCGTCACAAGTTCTCA
**LINK-ROR**	CTTCCAGGTCTCAGGAATGGG	AGGTGGCTGGTGAGAGATCC
**LIT1**	CAGGACATGCCCACACTAGC	CGTTTCCTCTTACAGTGGCGG
**MACC1**	GCCACACAGAGAGAAATACAAGG	CGTGCGACTAACTCTTGGCA
**MALAT1**	TAGCTTGGATCCTTGTGGGC	AACCCACCAAAGACCTCGAC
**MEG3**	GCCAGCTGTCCCTCTTACCT	GCATAGCAAAGGTCAGGGCT
**MIR17HG**	TGCCACGTGGATGTGAAGATT	GTTCTCCAGGAAGTTGCAGGC
**MMP7**	AAGTGGTCACCTACAGGATCG	GGGATCTCTTTGCCCCACAT
**MYC**	CAGGACCCGCTTCTCTGAAA	CTAACGTTGAGGGGCATCGT
**PCAT1**	AGGCCAAGCTACAGGGAGAAA	TGGCATTATCTTGGGAGGTTCC

**PCAT6**	CTCTTGGACAACACTCCGCC	ATCTGGCGCTGCACAAATGA
**PPIA**	ACTGAGTGGTTGGATGGCAAGC	AGCGCTCCATGGCCTCCACA
**PTENP1**	CATTCTTGCATGTATTTGGGTTAGG	GGTATATGGTCCAGAGTCCAGC
**TRIP13**	AGCTGAATGAAGATGGCCCC	TACCAAGCTGTCCCAAAGCC
**TUG1**	CTTTCACCATGGGGTGCCTG	AGCTGCTTTACACTGGGTGC
**UCA1**	TGCACCCTAGACCCGAAACT	CAAGTGTGACCAGGGACTGC
**WRAP53**	ACCGGACTGTGCGTGTTTTT	CTGGGCTGAAGGCTATGCAG
**ZEB2AS1**	ACGCGCCACCTATCTTTGTG	TGCCCTAAGATGCAGCTCCC

PCR primers designed for selected lncRNAs, mRNAs and housekeeping genes.

Supplemental Table 10: GEO datasets.

GEO datasets	Samples
GSE10715	peripheral blood (19 tumors vs 11 controls), blood smears (10 tumors vs 10 controls)
GSE13294	fresh-frozen primary tumor tissues (74)
GSE14333	fresh-frozen primary tumor tissues (290)
GSE17538	fresh-frozen primary tumor tissues (232)
GSE18088	frozen or formalin-fixed and paraffin-embedded tissues (53)
GSE18105	cancer tissues and normal adjacent tissues (17), laser capture microdissected primary tumors (77)
GSE20916	distant full-thickness normal colon, normal colon mucosa dissected from tumor, adenoma, and carcinoma (40)
GSE2109	tumor tissues (315)
GSE21510	laser capture microdissected and homogenized tumors (148)
GSE23878	tumor tissues (35 tumors vs 24 matched controls)
GSE33114	fresh frozen or formalin-fixed and paraffin-embedded tissues (90 tumors vs 6 controls)
GSE35896	primary tumor tissues (62)
GSE3629	53 ulcerative colitis tissues (10 UC-associated cancer, 43 non-cancer), 60 sporadic cancer tissues
GSE39582	fresh-frozen primary tumor tissues (566)
GSE41258	primary colon adenocarcinomas, adenomas, metastasis and corresponding normal mucosae (390)

GEO datasets on CRC samples analysed to confirm DE lncRNA expression. For each dataset, the type and number of samples is shown.
